# High-Sensitivity Sensor for Palladium Detection in Organic Solvent

**DOI:** 10.3390/ijms26125613

**Published:** 2025-06-11

**Authors:** Adrianna Pach, Agnieszka Podborska, Magdalena Luty-Błocho

**Affiliations:** 1 AGH University of Krakow, Faculty of Non-Ferrous Metals, Al. A. Mickiewicza 30, 30-059 Krakow, Poland; apach@agh.edu.pl; 2 AGH University of Krakow, Academic Centre for Materials and Nanotechnology, Al. A. Mickiewicza 30, 30-059 Krakow, Poland; podborsk@agh.edu.pl

**Keywords:** Pd(II) ion determination, azo-dye complexes, ethanol medium, UV–Vis spectrophotometry, fluorescence spectroscopy

## Abstract

A tandem UV–Vis and fluorescence spectroscopy method was developed for the detection of Pd(II) ions in ethanol. The formation of a complex between Pd(II) ions and tropaeolin OO (TR OO) is accompanied by a change in the color of the solution and evolution of the characteristic UV–Vis as well as fluorescence spectra. The optimal detection conditions were achieved at a 3:1 (mL/mL) volume ratio of Pd(II) to TR OO, at 50 °C. UV–Vis spectroscopy enabled the detection of complex formation process over time, while fluorescence spectroscopy allowed a rapid response within 10 min. The limit of detection (LOD) of Pd(II) ions using UV–Vis spectrophotometry was 10 μmol/dm^3^ at 535 nm. For spectrofluorimetric detection, the LOD was 10 μmol/dm^3^, with an emission signal observed at 630 nm after 10 min. The kinetics studies showed a stepwise complex formation pathway, supported by DFT calculations. The performance of the method was verified in the presence of interfering metal ions, including Li(I), Na(I), Al(III), Ni(II), Mg(II), Ca(II), Co(II), and Zn(II), confirming its applicability in complex matrices. This approach provides efficient palladium determination in organic solvents, contributing to sustainable practices in metal recycling.

## 1. Introduction

Palladium (Pd) contamination in organic reaction media is a well-known challenge in synthetic chemistry, due to the widespread use of Pd-catalyzed reactions in pharmaceutical, agrochemical, and materials-related applications. Reactions such as Suzuki-Miyaura, Heck, or Buchwald–Hartwig couplings are often carried out in organic solvents such as ethanol, methanol, or dimethylformamide, which help to dissolve reagents and stabilize the catalyst during the process [1,2,3,4]. However, even after standard purification steps, significant amounts of palladium can remain in the reaction mixture or final product. This metal is often present in solution in ionic form and can also exist as fine particles or colloids [5,6]. Palladium is also widely used as a catalyst in the automotive industry. In addition, it plays a role in alternative energy systems such as fuel cells, where it is used as a catalyst in electrochemical processes. In particular, it is employed in alcohol fuel cells, where it serves as a catalyst in ethanol (DEFC) or methanol (DMFC) oxidation processes [7,8,9]. Palladium from such applications can also enter the environment or reaction media as a contaminant, including organic solutions. The presence of residual palladium is of particular concern in pharmaceutical manufacturing, where even trace amounts can interfere with biological assays or pose toxicological risks. Exposure to palladium has been associated with various adverse health effects in humans, including respiratory issues, skin irritation, and allergic reactions [10,11,12,13]. As a result, international regulations have been established to control the levels of metal impurities in drug substances and products [1,14]. For this reason, the accurate determination of this element in pharmaceutical waste is important. Furthermore, due to its limited availability and high cost, the loss of palladium in waste streams is not only an environmental concern but also a significant economic issue, highlighting the need for reliable and efficient detection methods in complex organic systems.

The growing demand for palladium, especially in processes that use organic solvents, has increased the importance of developing effective recycling strategies. An essential step in this process is the ability to accurately detect palladium in complex organic samples. However, the determination of Pd(II) in such media remains challenging due to its typically low concentrations and potential interference from other metal ions [15]. Conventional analytical methods, including inductively coupled plasma mass spectrometry (ICP-MS) [16,17,18,19], inductively coupled plasma atomic emission spectroscopy (ICP-AES) [20,21,22], electrothermal atomic absorption spectroscopy (ETAAS) [23,24], and X-ray fluorescence spectrometry (XRF) [25,26], offer high sensitivity and precision, but they often require laborious sample preparation and the use of expensive instrumentation and can suffer from spectral interference [27,28]. In contrast, UV–Vis spectrophotometry and spectrofluorometry have gained attention as simpler and more accessible alternatives. These techniques provide fast, cost-effective, and sensitive analyses and are particularly useful for the detection of metal ions at trace levels [29,30]. In spectrophotometric methods, Pd(II) is typically converted into a colored coordination complex, which can be measured both qualitatively and quantitatively using UV–Vis spectrophotometry. Among the organic ligands used in these methods, azo-dyes play a key role due to their strong optical properties and their ability to form stable, colored complexes with transition metal ions, including Pd(II) [31,32]. In addition, combining UV–Vis spectrophotometry with spectrofluorometric analysis significantly enhances the applicability of the method, allowing for highly selective and sensitive detection of palladium, even in the presence of interfering elements.

Several recent studies have reported various types of palladium sensors, including electrochemical sensors, molecular probes, and fluorescent turn-on systems. For instance, electrochemical sensors such as adsorptive stripping voltammetry offer very low detection limits but often rely on mercury electrodes and are susceptible to surface contamination [33]. Carbon paste electrodes modified with ligands like thioridazine provide sensitivity but require acidic media and precise electrode preparation [34]. Fluorescent sensors based on Pd(0)-catalyzed reactions, such as allyl-deprotection [35] or nanoparticle-induced dehalogenation of fluorophores [36], can achieve high sensitivity and rapid detection; however, they frequently require strong reducing agents, elevated temperatures, or synthetically demanding probes. These limitations reduce their practical applicability in simple laboratory or industrial settings. This underscores the necessity of effective and accessible alternative, solvent-compatible methods. This supports the development of the current tandem UV–Vis and fluorescence-based approach utilizing commercially available azo-dyes in organic solvents. The goal of this study is to establish an effective method for detecting Pd(II) ions in organic media, with particular emphasis on ethanol-based systems commonly employed in palladium-catalyzed processes.

Based on our previous research, in which spectrophotometric methods using methyl orange and tropaeolin OO were successfully applied for Pd(II) detection in aqueous solutions (Luty-Błocho et al. [37]; Pach et al. [38]), we extend this approach by integrating UV–Vis spectrophotometry with spectrofluorometric analysis. The combination of these two techniques improves selectivity and sensitivity, allowing accurate detection of Pd(II) even at low concentrations and in the presence of potentially interfering metal ions such as Na(I), Al(III), Mg(II), Zn(II), Co(II), Ni(II), Li(I), and Ca(II). Tropaeolin OO was chosen as a complexing agent due to its excellent solubility in ethanol and its ability to form a distinct colored complex with Pd(II). The developed tandem method offers a promising tool for the determination of palladium in organic matrices, supporting efforts to improve analytical monitoring, metal recovery, and sustainable management of this critical resource.

## 2. Results and Discussion

### 2.1. Experimental Conditions

The determination of Pd(II) ions was carried out by UV–Vis spectrophotometry and spectrofluorometry under various experimental conditions, as presented in the Supplementary Materials, Table S1. To optimize the complex formation between Pd(II) ions and tropaeolin OO (TR OO) in ethanol, the effect of different volume ratios of Pd(II) and TR OO was investigated spectrophotometrically. The initial concentrations of both reagents were kept constant at 5 × 10^−5^ mol/dm^3^ and the total volume of each reaction mixture was 4 mL. To evaluate the influence of temperature, the experiments were conducted at two temperatures, 20 °C and 50 °C, using a TR OO stock solution aged for one month. After determining the optimal conditions for complex formation, spectrofluorometric measurements were performed. The fluorescence study was carried out under the previously optimized conditions, using excitation at 423 nm and recording emission spectra in the range of 520–800 nm. In addition, the effect of coexisting metal cations was investigated to evaluate the selectivity of Pd(II) ion determination. Cations such as Li^+^, Na^+^, Al^3+^, Ni^2+^, Mg^2+^, Ca^2+^, Co^2+^, and Zn^2+^ were introduced into the reaction mixtures and their influence on the spectrophotometric and spectrofluorometric response of the Pd(II) complex was studied.

### 2.2. Spectra of Reagents

Analysis of the primary UV–Vis spectra of the regents is an important aspect of ongoing research on the formation of metalorganic complexes between azo-dyes and Pd(II) ions. Characterization of the individual reagents is crucial for comparison with the spectra of the synthesized complexes. This avoids potential overlap between substrate and product spectra.

The UV–Vis spectra and corresponding molar coefficients of Pd(II) ions in ethanol are presented in the Supplementary Materials, Figure S1a,b, and summarized in Table 1. The spectrum of Pd(II) in ethanol exhibits two strong absorption maxima at 215 nm and 243 nm, along with two additional, less intense peaks at 319 nm and 423 nm. The absorbance–concentration plots constructed for these wavelengths showed excellent linearity, with correlation coefficients of R^2^ = 0.999 (215 nm), R^2^ = 0.998 (243 nm), R^2^ = 0.986 (319 nm), and R^2^ = 0.986 (423 nm) (see the Supplementary Materials, Figure S1b). Theoretical spectra for different forms of Pd(II) ions in ethanol (EtO) were also calculated. Detailed analysis suggests that the solution might contain a mixture of different forms: [PdCl_4_]^2−^, PdCl_3_(EtO)]^2−^, and PdCl_2_(EtO)_2_]^2−^ (see the Supplementary Materials, Figure S1c). In the study, for simplicity, we considered the first one.

The TR OO in ethanol exhibits two characteristic absorption maxima at 270 nm and 424 nm, as shown in the Supplementary Materials, Figure S2a,b, and summarized in Table 2. Compared to the absorption maxima previously recorded for TR OO in deionized water and buffer solutions [38], these peaks are shifted toward longer wavelengths. This bathochromic shift suggests solvent-dependent changes in the structure of the dye, due to differences in solvent polarity. The measured spectrum for TR OO is consistent with the calculated spectrum. The peak at 424 nm corresponds to the HOMO-LUMO transition in the TR OO molecule. The peak at about 270 nm is associated with the HOMO-LUMO+1 transition (Supplementary Materials, Figure S2c,d). The calibration plots constructed for these wavelengths demonstrated excellent linearity, with correlation coefficients of R^2^ = 0.999 at both 270 nm and 424 nm (see the Supplementary Materials, Figure S2b).

To characterize the fluorescence properties of the individual reagents prior to complex formation, emission spectra of TR OO and Pd(II) solutions were recorded at a concentration of 5 × 10^−5^ mol/dm^3^ for both components at 20 °C (see the Supplementary Materials, Figure S3). Measurements were carried out using an excitation wavelength of 423 nm. The recorded fluorescence spectra indicate that neither Pd(II) ions nor TR OO in ethanol exhibit peaks in the emission spectrum. This allows the use of spectrofluorometry to observe the formation of the organometallic compound resulting from the reaction between the metal ions and the dye.

### 2.3. Optimalization and Characterization of Pd(II)—TR OO Complex Formation

**Figure 1:**
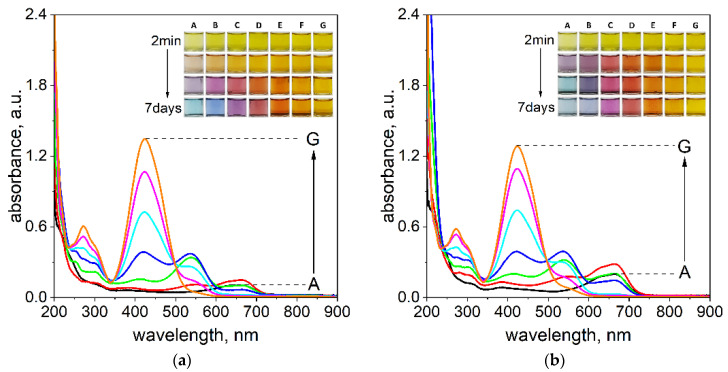
The UV–Vis spectra of solutions containing the mixture of Pd(II) ions with TR OO at different volume ratios of Pd(II) to TR OO: A—3.5:0.5; B—3.0:1.0; C—2.5:1.5; D—2.0:2.0, E—1.5:2.5; F—1.0:3.0; G—0.5:3.5 (mL/mL) at temperatures of 20 °C (**a**) and 50 °C (**b**). The UV–Vis was registered after 7 days in ethanol. Conditions: the value of concentration before reagent mixing: C_0,TR OO=Pd(II)_ = 5 × 10^−5^ mol/dm^3^, path length 1 cm. Assignment of recorded spectrum colors to samples: Sample A—black; B—red; C—green; D—dark blue; E—blue; F—pink and G—orange.

The formation of the Pd(II)–TR OO complex in ethanol was investigated using both UV–Vis spectrophotometry and fluorescence spectroscopy to determine the optimal process conditions. The complexation process at various volume ratios of the reactants was monitored spectrophotometrically at time intervals of 5 min, 1 h, 24 h, and 7 days at two temperatures, 20 °C and 50 °C (see Figure 1a,b and the Supplementary Materials, Figures S4 and S5). Based on the results obtained, fluorescence measurements were subsequently performed for the volume ratio that provided the most favorable complexation behavior. In all experiments, the initial concentrations of Pd(II) ions and TR OO were kept constant at 5 × 10^−5^ mol/dm^3^. A detailed description of the experimental parameters is provided in the Supplementary Materials, Table S1.

After mixing the reagents, the solutions initially exhibited a yellow color, corresponding to the native color of TR OO. After 1 h at a temperature of 20 °C, a slight shift in color was observed toward yellow-orange tones (see Figure 1a). As the reaction proceeded, more pronounced color changes developed, which were dependent on the volume ratio of the reagents. After 24 h, the solutions shifted to light purple (sample A), purple pink (sample B), pinkish (sample C), and orange (samples D and E), while samples F and G remained visually unchanged (see Figure 1a). After 7 days, further color transformations were observed: samples A and B developed a blue hue of varying intensity, sample C turned violet, sample D remained pinkish, sample E shifted toward orange, and samples F and G remained yellow (see Figure 1a).

The UV–Vis spectra were recorded at 5 min, 1 h, 24 h, and 7 days to monitor the progress of complex formation at 20 °C (see Figure 1a, Supplementary Materials, Figure S4a–d). Initially, three characteristic absorption maxima at 241 nm, 270 nm, and 424 nm were observed. The peaks at 270 nm and 424 nm originate from the original TR OO spectrum. At 20 °C, no significant spectral changes were observed during the early stages of the process. However, after 24 h, a pronounced decrease in the intensity of the initial peaks was observed in samples A–D, accompanied by the appearance of new maxima at 535 nm and 664 nm (see Figure 1a and the Supplementary Materials, Figure S4c). These spectral changes indicate the formation of the Pd(II)–TR OO complex. In sample E, only the peak at 535 nm was observed. In contrast, no significant spectral changes were detected in samples F and G. After 7 days, samples A–E showed a further decrease in the original absorption bands and a further increase in intensities at 535 nm and 664 nm (see Figure 1a and the Supplementary Materials, Figure S4d), confirming the progress of complex formation over time.

To accelerate the formation of the Pd(II)–TR OO complex, the process was also carried out at an elevated temperature of 50 °C (see Figure 1b, Supplementary Materials, Figure S5a–d). A comparison of visual observations and UV–Vis spectra revealed that the higher temperature significantly shortened the time required for complex formation. After 1 h of incubation at 50 °C, all tested samples showed color changes characteristic of those observed after 24 h at 20 °C (see Figure 1a). However, after 24 h at 50 °C, sample A turned blue, sample B turned dark purple, sample C changed to pink, and sample D turned red-orange. Samples E and F did not change their color after 24 h. After 7 days, the intensity of the colors in samples A–D became slightly lighter, indicating stabilization of the complex. The spectral data supported these visual changes. After 1 h, new absorption bands at 535 nm and 664 nm appeared in samples A–D, with varying intensities, indicating the formation of the Pd(II)–TR OO complex. These maxima continued to increase in intensity over time, as observed in spectra recorded after 24 h and 7 days (see Figure 1b and the Supplementary Materials, Figure S5b–d), confirming the progress of the complexation process. The most pronounced spectral response and the most visible color change of the solution over time were recorded for sample B (3.0 mL Pd(II) and 1.0 mL TR OO). This indicates the most efficient complex formation at this volume ratio.

To further confirm the validity of the choice of volume ratio for the complex under study, Job’s method was used. This technique is a useful approach for determining the stoichiometry of metal–ligand complexes. In this study, Job’s graph was constructed for the Pd(II)–TR OO complex formed at 50 °C and absorbance values at 535 nm and 664 nm (see the Supplementary Materials, Figures S6, S7a,b and S8a,b). These wavelengths correspond to the characteristic maxima of the formed complex. The results indicated that the most favorable volume ratio was 2.0 mL Pd(II) to 2.0 mL TR OO at 535 nm, and 3.0 mL Pd(II) to 1.0 mL TR OO at 664 nm after 24 h. Based on observations of color changes, registered spectra, and Job’s method, a volumetric ratio of 3.0 mL Pd(II) to 1.0 mL TR OO was selected for further study. Interestingly, these stoichiometries differ from those reported for Pd(II)–TR OO complexes formed in aqueous media, where a 2.5:1.5 volume ratio was observed [38]. This suggests that the mechanism and structure of complex formation are strongly influenced by the solvent environment.

**Figure 2:**
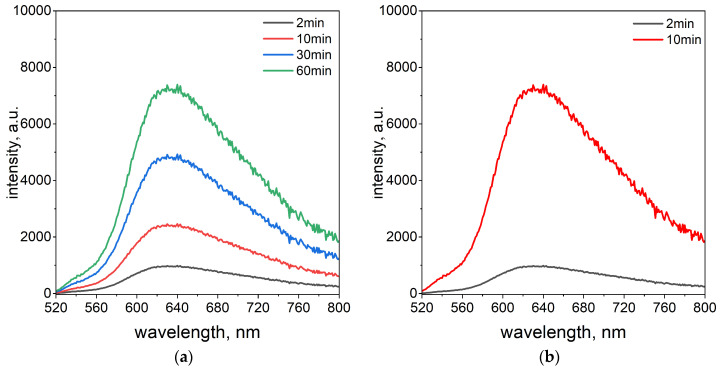
The fluorescence emission spectra solution containing the mixture of TR OO with Pd(II) ions at temperatures of 20 °C (**a**) and 50 °C (**b**) over time from 2 min to 60 min. Conditions: volumetric ratio, 3.0 mL Pd(II)/1.0 mL TR OO; the value of concentration before reagent mixing, C_0,TR OO,Pd(II)_ = 5 × 10^−5^ mol/dm^3^; path length, 1 cm.

In addition to the spectrophotometric studies, the fluorescence properties of the Pd(II)–TR OO complex were investigated. This analysis provided additional knowledge about the sensitivity of Pd(II) ions detection. The fluorescence properties were investigated using the same optimal volume ratio of reagents (3.0 mL Pd(II)/1.0 mL TR OO) as determined by UV–Vis analysis. Fluorescence emission spectra were recorded at an excitation wavelength of 423 nm at two temperatures, 20 °C and 50 °C (see Figure 2a,b).

The fluorescence measurements were conducted at 2, 10, 30, and 60 min intervals at a temperature of 20 °C. A gradual change in the emission spectrum was observed over time, with the fluorescence signal reaching a plateau after 60 min and exhibiting maximum emission at 630 nm (see Figure 2a). In contrast, at 50 °C, the fluorescence intensity reached its maximum just 10 min after the start of the measurement (see Figure 2b). The elevated temperature significantly accelerated the complexation process. This rapid increase confirms that the complex forms more efficiently at higher temperatures, in agreement with the results obtained from UV–Vis spectrophotometry (see Figure 1b). An important advantage of the spectrofluorimetric method is its ability to detect Pd(II)–TR OO complex formation much faster than the spectrophotometric technique. The difference in reading times between the two techniques stems from variations in their sensitivity.

### 2.4. Kinetics and Mechanism of Pd(II)–TR OO Complex Formation

**Figure 3:**
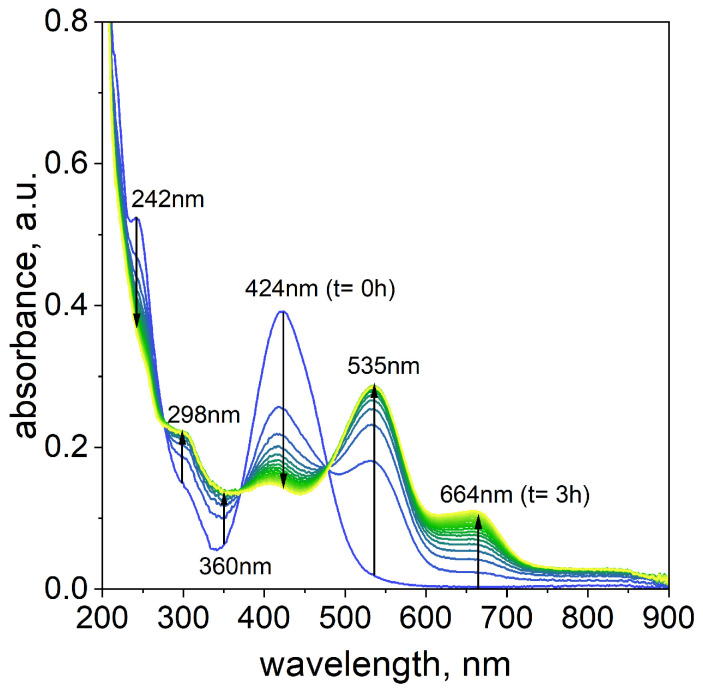
The UV–Vis spectra evolution of the solution containing the mixture of TR OO and Pd(II). Conditions: volumetric ratio, 3.0 mL Pd(II)/1.0 mL TR OO; the value of concentration of Pd(II) ions after mixing with TR OO, C_0,TR OO=Pd(II)_ = 5 × 10^−5^ mol/dm^3^; T = 50 °C.

The process of Pd(II)–TR OO complex formation was studied over time to better understand its kinetics and potential reaction pathway. The reaction was carried out at 50 °C using a volume ratio of 3.0 mL Pd(II) to 1.0 mL TR OO (see the Supplementary Materials, Table S1). The reaction progress was monitored by UV–Vis spectrophotometry for 180 min, with spectra recorded at 10 min intervals (see Figure 3).

Immediately after mixing the reagents, two characteristic absorption maxima were observed at 242 nm and 424 nm. In the 200–300 nm region, there was spectral overlap between Pd(II) ions (Supplementary Materials, Figure S1a) and TR OO (Supplementary Materials, Figure S2a), indicating contributions from both components. The most significant spectral changes associated with complex formation occurred in the 400–700 nm region.

Taking into account the change in the characteristic UV–Vis spectra in the wavelength range from 400 nm to 700 nm, it can be assumed that the Pd(II)–TR OO complex’s formation proceeds as follows:

(1) $$A\;\overset{k_{1}}{\to }\;B\;\overset{k_{2}}{\to }\;C$$

where

*A*—substrate with maximum at 424 nm;

*B*—intermediates with maximum at 535 nm;

*C*—final product with maximum at 664 nm;

*k*_1_, *k*_2_—rate constants for forming intermediate (*B*) and product (*C*), respectively.

The proposed reaction pathway suggests that A component, located at 424 nm, decreases in intensity over time. Simultaneously, increasing intensities of the new maxima at 535 and 664 nm were observed as the reaction progressed. In the reaction pathway (1), the new maxima suggest the formation of components B and C, which is related to the formation of intermediates and final products over time.

**Figure 4:**
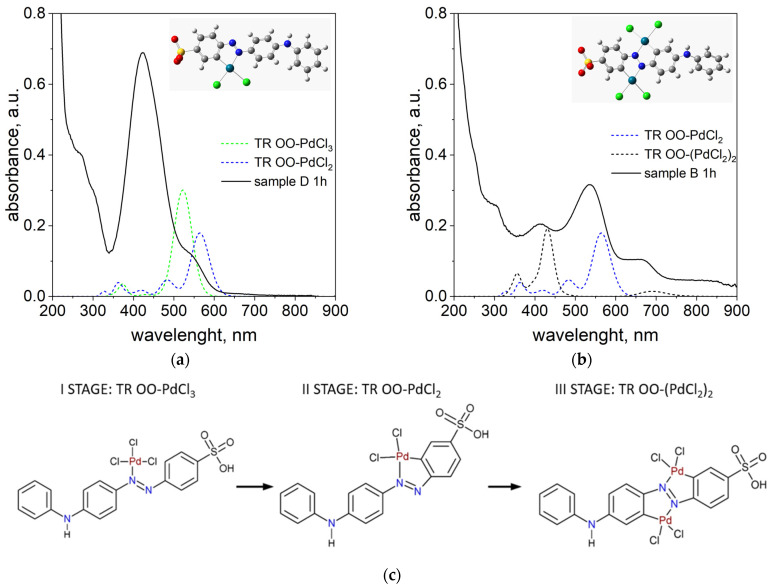
Proposed structure of the formed complex and comparison of the spectra measured for sample D and spectra calculated for the Pd(II)/TR OO complex, whose Pd(II)/TR OO ratio is 1:1 (**a**) and measured for sample B and calculated for the TR OO-(PdCl_2_)_2_ complex, in which the TR OO ratio to Pd is 1:2 (**b**), and the structures of TR OO–Pd complexes that form in solution during the process (**c**). Notation: grey—carbon; white—hydrogen; yellow—sulfur; red—oxygen; blue—nitrogen; dark green—palladium; green—chloride.

As proposed in our previous work [38], the appearance of a peak around 500 nm is attributed to the formation of Pd-N and Pd-C bonds within the phenyl ring of TR OO. To confirm this, DFT calculations were performed for model complexes containing 1 and 2 Pd atoms attached to one TR OO molecule, designated TR OO-PdCl_2_ and TR OO-(PdCl_2_)_2_, respectively. Theoretical spectra calculated for these complexes were compared with experimental spectra measured for solutions in which the Pd/TR OO ratio was 1:1 (sample D) and 3:1 (sample B) (see Figure 4a,b). Detailed analysis of theoretical and experimental data confirmed the multi-stage formation of the organometallic complex (see Figure 4c).

In the first stage, one Pd atom is attached to nitrogen atom in TR OO and the bond Pd-N is formed. The TD-DFT calculated spectrum for the TR OO-PdCl_3_ complex has a peak with a maximum at 522 nm (Figure 4a).

In the second step, a bond is formed between palladium, which is bonded to the nitrogen atom, and the carbon atom in the benzene ring, and the maximum of the peak shifts towards longer wavelengths. The theoretical spectrum calculated for a new TR OO-PdCl_2_ complex has an intense peak with a maximum of 563 nm. In all of these complexes, the Pd/TR OO ratio is 1:1. Therefore, the spectra calculated for these complexes were compared with the spectrum measured for sample D, in which the Pd/TR OO ratio was also 1:1 (Figure 4a). In the experimental spectrum, we see an intense peak with max at 422 nm, which comes from the substrates, and a new peak above 500 nm appears, coming from the tropaeolin–palladium complexes formed in the solution. The peak at approximately 535 nm is also very clearly visible in the spectrum measured for sample B (Pd/TR OO = 3:1) measured after 1 h.

In the next step, the second Pd atom is connected, and a peak around 660 nm appears in the experimental spectrum, which is consistent with the peak (686 nm) in the theoretical spectrum calculated for the TR OO–(PdCl_2_)_2_ complex. If we follow the changes in the spectrum over time for sample B presented in Figure 3, we will see full compliance with the proposed mechanism. The peaks originating from the substrates at 242 nm and 424 nm disappear or are shifted (from 424 to 415 nm), and new peaks originating from palladium–tropaeolin complexes are formed: 298, 360, 535, and 664 nm. The theoretical spectra are not in perfect agreement with the experimental spectra, which is due to the fact that the calculations are carried out for a single molecule of the complex, while in the solution we have several coexisting forms whose spectra overlap (see the Supplementary Materials, Figure S9).

### 2.5. Determining the Limit of Detection of the Pd(II) Ions for the Pd(II)–TR OO Complex

**Figure 5:**
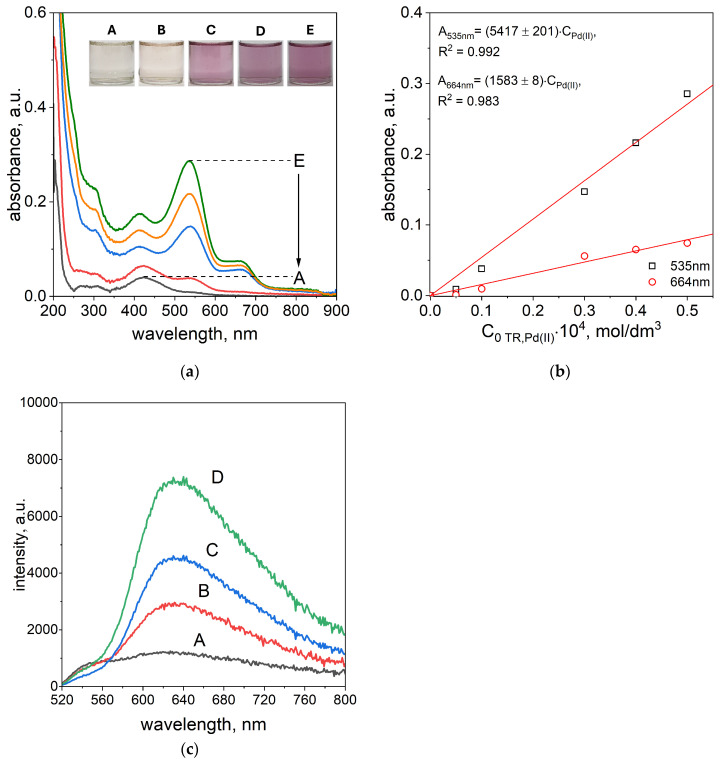
Spectra of solutions containing mixture of TR and Pd(II) ions after 1 h (**a**); dependency of absorbance vs. initial concentration of Pd(II) ion after 1 h, A: 5 × 10^−6^ mol/dm^3^; B: 1 × 10^−5^ mol/dm^3^; C: 3 × 10^−5^ mol/dm^3^; D: 4 × 10^−5^ mol/dm^3^, E: 4 × 10^−5^ mol/dm^3^. Assignment of recorded spectrum colors to samples: Sample A—black; B—red; C—blue; D—orange; E—dark green. (**b**) Fluorescence emission spectra registered after 10 min A: 5 × 10^−6^ mol/dm^3^; B: 1 × 10^−5^ mol/dm^3^; C: 3 × 10^−5^ mol/dm^3^; D: 5 × 10^−5^ mol/dm^3^ (**c**). Conditions: T = 50 °C, path length 1 cm.

Determining the limit of detection (LOD) is a critical step in validating the performance of newly developed methods for metal ion detection. The LOD is an indicator of the sensitivity of the method and its potential for practical application. In this study, the LOD for Pd(II) ions was assessed using both UV–Vis spectrophotometry and fluorescence spectroscopy (see Figure 5a,c and the Supplementary Materials, Figure S10a,b). The measurements were conducted in ethanol at 50 °C, using Pd(II) and TR OO solutions at concentrations ranging from 5 × 10^−6^ mol/dm^3^ to 5 × 10^−5^ mol/dm^3^. The analysis was carried out at the optimal volume ratio of Pd(II) to TR OO, established as 3:1.

The UV–Vis spectra of Pd(II)–TR OO complexes obtained after 5 min, 1 h, and 24 h for varying Pd(II) concentrations (from A: 5 × 10^−6^ mol/dm^3^, B: 1 × 10^−5^ mol/dm^3^, C: 3 × 10^−5^ mol/dm^3^, D: 4 × 10^−5^ mol/dm^3^, E: 5 × 10^−5^ mol/dm^3^), along with the corresponding solution colors, are presented in Figure 5a and the Supplementary Materials, Figure S10a,b. The color of the solutions after 1 h was dependent on the concentration of the reagents. At the lowest concentrations, the solutions appeared nearly colorless (sample A) or slightly pink (sample B), while at higher concentrations, samples C, D, and E showed a distinct pink color (see Figure 5a). After 24 h, the color of samples A and B remained unchanged, whereas samples C, D, and E turned violet (see the Supplementary Materials, Figure S10b). The UV–Vis spectrum of sample A after 1 h showed a single absorption maximum at 424 nm, originating from the native spectrum of TR OO. In sample B, a new absorption peak appeared at 535 nm, indicating the formation of the Pd(II)–TR OO complex. In samples C, D, and E, two characteristic absorption maxima were observed at 535 nm and 664 nm, confirming the presence of the fully formed complex (see Figure 5a). After 24 h, the spectra of samples A and B remained unchanged, while absorbance at 664 nm increased in samples C, D, and E, indicating continued formation and stabilization of the complex over time (see the Supplementary Materials, Figure S10b).

The absorbance intensity increased with increasing reagent concentrations. Based on these spectra, a graph representing absorbance vs. initial Pd(II) concentration was constructed (see Figure 5b and the Supplementary Materials, Figure S10c). From this, molar absorption coefficients were determined for the characteristic wavelengths, enabling quantitative estimation of Pd(II) in solution. After 1 h of reaction, Pd(II) concentrations could be determined using absorbance at 535 nm (samples B–E) and at both 535 and 664 nm for samples C, D, and E. The corresponding calibration curves showed high linearity, with correlation coefficients (R^2^) of 0.992 at 535 nm and 0.983 at 664 nm. After 24 h, the R^2^ values remained slightly lower but still high, at 0.989 for 535 nm and 0.985 for 664 nm (see the Supplementary Materials, Figure S10c).

The fluorescence emission spectra of the Pd(II)–TR OO complex at varying Pd(II) concentrations, recorded after 10 min at 50 °C, are presented in Figure 5c. Similar to the UV–Vis results, the fluorescence intensity increased with rising Pd(II) concentration. Increased emission was observed in the range of 520–800 nm, confirming complex formation between Pd(II) and TR OO, as discussed in the previous paragraph. A well-defined fluorescence maximum appeared at approximately 630 nm, with the most intense signals recorded for samples B–D.

Based on the obtained results, it can be concluded that the combination of UV–Vis spectrophotometry and fluorescence spectroscopy provides high sensitivity for the detection of Pd(II) ions using the Pd(II)–TR OO system. In the case of the spectrophotometric method, Pd(II) ions could be detected at concentrations as low as 1 × 10^−5^ mol/dm^3^ (1.06 ppm) after 1 h, corresponding to an estimated LOD of 10 μmol/dm^3^ when using the absorption maximum at 535 nm. Additionally, detection at two wavelengths (535 nm and 664 nm) enabled the identification of Pd(II) at concentrations of 3 × 10^−5^ mol/dm^3^ (3.19 ppm) and above. In comparison, the spectrofluorimetric approach demonstrated an even faster response and high sensitivity, allowing for the detection of Pd(II) ions at concentrations as low as 1 × 10^−5^ mol/dm^3^ (1.06 ppm) within just 10 min.

Detection limits reported for various palladium determination methods vary widely depending on the analytical technique and sample matrix. Flame atomic absorption spectrometry (FAAS) combined with deep eutectic solvent-based liquid phase microextraction (DES-LPME) and single drop microextraction (SQT) enables the detection of Pd at 7.4 µg/L (0.0074 ppm) in environmental water samples [39]. Traditional AAS provides an LOD of 0.10 mg/L (0.10 ppm) for pharmaceutical bulk materials digested in nitric acid [40], while high-resolution continuum source electrothermal AAS (HR-CS ETAAS) achieves 0.19 mg/kg (0.19 ppm) in automotive catalyst matrices [41]. Capillary electrophoresis (CE) with an online flow manifold allows for detection at 2.1 µg/L (0.0021 ppm) in water [42]. Flow-injection spectrophotometry (DAD, 410 nm) yields an LOD of 0.1 mg/L (0.1 ppm) for solid matrices including road dust, ores, and catalytic converters [43]. Ion chromatography coupled with ICP-MS achieves ultra-trace detection levels of 230 ng/L (0.00023 ppm) in environmental samples such as road dust and atmospheric particles [44]. Electrochemical detection using screen-printed carbon electrodes modified with gold nanoparticles (AuNPs/SPCE) reaches 0.1 µg/L (0.0001 ppm) in road dust samples [45]. Although these techniques demonstrate excellent sensitivity, they often require costly instrumentation, complex sample treatment, or hazardous reagents. In contrast, the spectroscopic method presented here, based on UV–Vis and fluorescence detection using a commercially available azo-dye in ethanol, offers a practical alternative that balances ease of use, speed, and accessibility with adequate sensitivity for analysis in organic systems.

### 2.6. The Influence of Metal Cations

**Figure 6:**
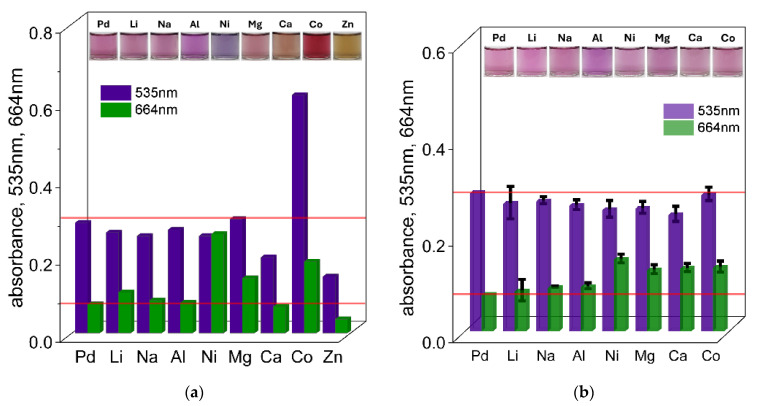
The value of absorbance at wavelengths of 535 nm and 664 nm for solutions containing Pd(II) ions with TR OO and Pd(II)–TR OO with metal cations—Li^+^, Na^+^, Al^3+^, Ni^2+^, Mg^2+^, Ca^2+^, and Co^2+^—at concentrations of 0.1 mol/dm^3^ (**a**) and 0.01 mol/dm^3^ (each measurement was performed in triplicate (n = 3), error bars represent standard deviation) (**b**), after 1 h in ethanol. Conditions: C_0,TR OO_ = 1.25 × 10^−5^ mol/dm^3^, C_0,Pd(II)_ = 3.75 × 10^−5^ mol/dm^3^, volumetric ratio mixing of Pd(II) ions and TR OO = 3.0 mL: 1.0 mL, T = 50 °C.

An important aspect of Pd(II) ion determination is the ability to perform selective detection in the presence of high concentrations of coexisting metal cations. These cations are often present together with palladium in industrial wastes. To investigate the selectivity of the proposed system, complex formation between Pd(II) and TR OO was studied using a combination of UV–Vis spectrophotometry and fluorescence spectroscopy in the presence of various metal cations, including Li^+^, Na^+^, Al^3+^, Ni^2+^, Mg^2+^, Ca^2+^, Co^2+^, and Zn^2+^. These interfering ions were introduced as their chlorate salts, in accordance with our previous studies [38], at concentrations of 0.1 mol/dm^3^ and 0.01 mol/dm^3^. The concentrations of Pd(II) and TR OO were maintained at a constant level of 1 × 10^−5^ mol/dm^3^, with a constant volume ratio of 3 mL of Pd(II) ions to 1 mL of TR OO. All measurements were performed at 50 °C. Based on these spectra, the intensity of the signal (absorbance value) at characteristic wavelengths (535 and 664 nm) at different times (1 h and 24 h) (see Figure 6a,b) was compiled for samples containing different metal cations at concentrations of 0.1 and 0.01 mol/dm^3^ (see the Supplementary Materials, Figures S11a–i and S12a–h).

As a first step, the possibility of detecting Pd(II) ions in the presence of high concentrations (0.1 mol/dm^3^) of interfering cations was investigated. The UV–Vis spectra with the solution colors recorded after 5 min, 1 h, and 24 h are shown in Figure 6a and the Supplementary Materials, Figure S11a–i. After mixing Pd(II) and TR OO in the presence of different metal cations, most of the solutions initially appeared yellow. However, some deviations were observed depending on the added ion: solutions containing Ni(II) showed a greenish color, Co(II) produced an orange color, and Zn(II) resulted in a yellow-brown color. After 1 h, solutions containing Na^+^ and Li^+^ ions turned pink, corresponding to the color of the Pd(II)–TR OO complex, allowing qualitative detection of Pd(II) in solutions. In contrast, the presence of other cations made qualitative identification more difficult. For example, the solution containing Al(III) developed a pink-purple color, while Ni(II) produced a purple hue. Significant deviations from the characteristic complex color were also observed for Mg(II), Ca(II), Co(II), and Zn(II), which became pink-orange, brown-orange, light red, and dark yellow, respectively. Further changes in solution color were observed after 24 h (see the Supplementary Materials, Figures S11a–i and S13a). The reference sample containing only Pd(II) ions developed a dark purple color. Only the solutions containing Li(I) and Mg(II) turned light purple with varying intensity. The Na(I) solution remained pink, while the Ca(II) sample became pale pink. Notably, the Al(III) and Ni(II) solutions turned blue, while the Co(II) solution retained its bright red color.

The recorded UV–Vis spectra showed that in the presence of Li(I), Na(I), and Al(III), the absorbance values at both 535 nm and 664 nm remained similar to those observed for the Pd(II)–TR OO complex alone (see Figure 6a and the Supplementary Materials, Figure S11a–i). This indicates that qualitative detection of Pd(II) is possible in the presence of high concentrations of these cations. In the presence of Mg(II), detection was also possible at a single wavelength of 535 nm, while in the case of Ca(II), a detectable response was observed at 664 nm after 1 h. In contrast, significant differences in absorbance were observed for samples containing Ni(II), Ca(II), Co(II), and Zn(II). The most pronounced interference was caused by Zn(II), which showed a significantly different spectrum, consistent with the distinct color changes observed visually. After 24 h, the absorbance of the sample containing only Pd(II) increased at both 535 nm and 664 nm (see the Supplementary Materials, Figures S11a–i and S13a). However, noticeable spectral changes were observed in solutions containing other metal ions, especially in samples containing Co(II), Ni(II), and Zn(II). Under these conditions, Pd(II) could only be detected in the presence of Li(I) and Na(I), using the absorbance at 535 nm. For other cations, the absorbance values at both 535 nm and 664 nm differed significantly from those of the pure Pd(II)–TR OO complex, making Pd(II) determination impossible after 24 h in the presence of high concentrations of these interfering ions.

The differences in solution color and UV–Vis spectra observed for samples containing Al(III), Co(II), Ni(II), and Zn(II) suggest that these cations are capable of forming stable complexes with azo-dyes [46,47]. The different colors and registered spectra, when compared to the Pd(II)–TR OO complex, indicate a higher affinity of these metal ions for the azo-ligand. In particular, the noticeable deviations in solution color further support the formation of competing metal–dye complexes. The spectrophotometric data confirm that, at high concentrations, these cations may form more favorable or faster complexes with TR OO in ethanol, which can hinder or even prevent the detection of Pd(II) under the tested conditions.

The determination of Pd(II) ions in the presence of other metal cations at concentrations of 0.1 mol/dm^3^ was too high for Pd(II) detection in the presence of most interfering ions. Therefore, a lower concentration of 0.01 mol/dm^3^ was used to improve the selectivity of the method. After mixing the reagents, most of the solutions containing the tested cations initially appeared yellow, with the exception of the Zn(II)-containing sample. After 1 h, almost all solutions developed a pink color similar to that of the Pd(II)–TR OO complex (see Figure 6b and the Supplementary Materials, Figure S12a–h), allowing qualitative detection of Pd(II) in these solutions. The only exception was the Zn(II) solution, which showed a different color response, suggesting a problem in the complexation process. After 24 h (see the Supplementary Materials, Figures S12a–h and S13b), the solutions containing Li(I), Ni(II), Ca(II), and Mg(II) retained colors comparable to the Pd(II)–TR OO complex, gradually shifting towards a purple hue. The solutions containing Co(II) and Na(I) ions became pink-purple, suggesting a possible effect on the complex formation process that should be considered in the qualitative analysis. In addition, a slight precipitate was observed in some samples. The most significant deviation was observed for the Al(III)-containing solution, which changed to light blue—similar to the behavior observed at the higher concentration of 0.1 mol/dm^3^. This indicates a strong interaction between Al(III) ions and TR OO, which significantly interferes with the visual detection of Pd(II) ions.

The optical properties and their changes during the complex formation process were also monitored spectrophotometrically, and the results are shown in Figure 6b and the Supplementary Materials, Figure S12a–h. When the concentration of interfering metal cations was reduced to 0.01 mol/dm^3^, the recorded UV–Vis spectra showed significant differences compared to those obtained at higher cation concentrations (see Figure 6a). Each measurement was performed three times to ensure repeatability, and the standard deviations are represented by black error bars in the plots. In the UV–Vis spectra recorded after 1 h for all cations (except Zn(II)), the absorbance values at both 535 nm and 664 nm were nearly identical to those of the Pd(II)–TR OO complex, indicating minimal interference with complex formation under these conditions. After 24 h (see the Supplementary Materials, Figures S12a–h and S13b), the absorbance for samples containing Li(I), Na(I), Mg(II), Ca(II), and Co(II) remained similar to that of the reference complex at both wavelengths, suggesting that these cations had a minimal effect on the stability or progress of complex formation. In contrast, solutions containing Al(III) and Ni(II) showed pronounced deviations in absorbance at both 535 nm and 664 nm. Among them, Al(III) caused the most significant spectral shift, consistent with previous observations and indicating strong interference with the Pd(II)–TR OO complex.

**Figure 7:**
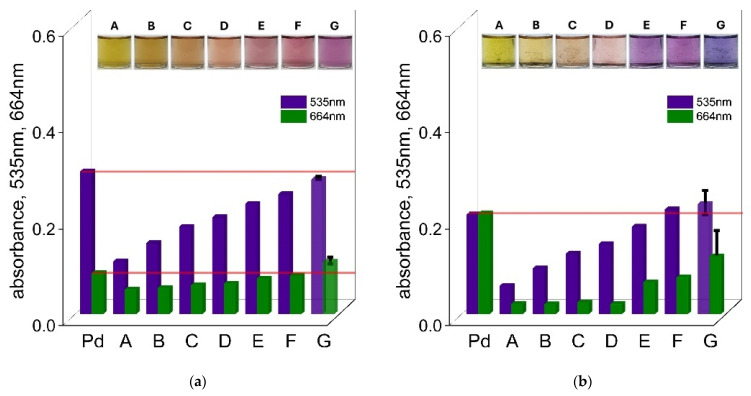
The values of absorbance at wavelengths of 535 nm and 664 nm for solutions containing Pd(II) ions, TR OO, and Zn^2+^, at different concentrations of Zn^2+^—A: 1 × 10^−1^ mol/dm^3^; B: 5 × 10^−2^ mol/dm^3^; C: 1 × 10^−2^ mol/dm^3^; D: 5 × 10^−3^ mol/dm^3^; E: 1 × 10^−3^ mol/dm^3^; F: 5 × 10^−4^ mol/dm^3^; G: 1 × 10^−4^ mol/dm^3^ (measurement was performed in triplicate (n = 3); error bars represent standard deviation)—after 1 h (**a**) and 24 h (**b**) in ethanol. Conditions: C_0,TR OO_ = 1.25 × 10^−5^ mol/dm^3^, C_0,Pd(II)_ = 3.75 × 10^−5^ mol/dm^3^, volumetric ratio mixing of Pd(II) ions and TR OO = 3.0 mL: 1.0 mL, T = 50 °C.

In the case of Zn(II) ions, reducing the concentration from 0.1 mol/dm^3^ to 0.01 mol/dm^3^ was still not enough for accurate detection of Pd(II), as shown in Figure 7a,b. The solutions remained yellow after 1 h, indicating strong interference in the complexation process. These results indicate the formation of a stable Zn(II)–TR OO complex. By further decreasing the Zn(II) concentration to 0.0001 mol/dm^3^, the color of the solution changed to pink after 1 h and to violet after 24 h, consistent with the color of the Pd(II)–TR OO complex (see Figure 7a,b and the Supplementary Materials, Figure S14a–c). The corresponding UV–Vis spectra confirmed that this significant decrease in Zn(II) concentration allowed the successful detection of Pd(II) at 535 nm and 664 nm after 1 h (see Figure 7a) and at 535 nm after 24 h (see Figure 7b). To confirm the repeatability of these results, the lowest concentration measurements were repeated three times, and the standard deviations are shown as black error bars in the graphs.

**Figure 8:**
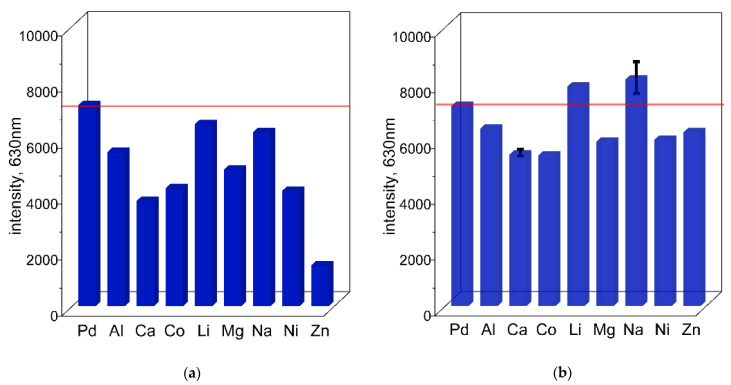
The value of fluorescence obtained at 630 nm for solutions containing TR OO with Pd(II) ions and metal cations—Al^3+^, Ca^2+^, Co^2+^, Li^+^, Mg^2+^, Na^+^, Ni^2+^, and Zn^2+^—at concentrations of 0.1 mol/dm^3^ (**a**) and 0.01 mol/dm^3^ (0.0001 mol/dm^3^ for Zn^2+^) (**b**), after 10 min in ethanol. Conditions: C_0,TR OO_ = 1.25 × 10^−5^ mol/dm^3^, C_0,Pd(II)_ = 3.75 × 10^−5^ mol/dm^3^, volumetric ratio mixing of Pd(II) ions and TR OO = 3.0 mL: 1.0 mL, T = 50 °C.

The possibility of detecting Pd(II) ions in the presence of different metal cations was also evaluated using spectrofluorometry. Similar to the spectrophotometric approach, the formation of the Pd(II)–TR OO complex was studied in the presence of Li(I), Na(I), Al(III), Ni(II), Mg(II), Ca(II), Co(II), and Zn(II) at concentrations of 0.1 mol/dm^3^ and 0.01 mol/dm^3^ (and for Zn(II) also at 0.0001 mol/dm^3^), at 50 °C (Figure 6 and Figure 7a,b and the Supplementary Materials, Figures S11 and S12). Fluorescence measurements were performed using an excitation wavelength of 423 nm, and in each case the maximum emission was observed at 630 nm after just 10 min (see Section 2.3). The results are presented as bar graphs showing the relative emission intensity of Pd–TR OO with metal cations compared to the single Pd(II)–TR OO complex (see Figure 8a,b), together with the corresponding spectral data (Supplementary Materials, Figures S15a–i and S16a–h). At the higher cation concentration (0.1 mol/dm^3^), significant quenching of Pd(II)–TR OO fluorescence was observed in the presence of Al(III), Ni(II), Mg(II), Ca(II), and Co(II) (see Figure 8a and the Supplementary Materials, Figure S15a–i). In particular, Zn(II) showed a strong fluorescence quenching effect. In contrast, Li(I) and Na(I) maintained fluorescence intensities similar to that of the Pd(II)–TR OO complex alone, consistent with previous UV–Vis results showing that high concentrations of these metal ions do not significantly interfere with complex formation. When the cation concentration was reduced to 0.01 mol/dm^3^, fluorescence quenching remained evident for Al(III), Ni(II), Mg(II), Ca(II), and Co(II) (see Figure 8b and the Supplementary Materials, Figure S16a–l). Even at a significantly reduced Zn(II) concentration (0.0001 mol/dm^3^), a quenching effect was still present, although less pronounced than at higher concentrations. On the other hand, decreasing the concentration of Li(I) and Na(I) resulted in an increase in fluorescence intensity, with an emission close to or slightly exceeding that of the pure Pd(II)–TR OO complex.

## 3. Materials and Methods

### 3.1. Chemicals

*Tropaeolin OO (Sodium 4-[(E)-(4-anilinophenyl)diazenyl]benzene-1-sulfonate).* A stock solution was prepared by dissolving 0.01 g of azo-dye powder (Merck, Darmstadt, Germany) in 100 mL of ethanol resulting in a concentration of 2.6 × 10^−4^ mol/dm^3^. Then, the respective volume of the stock solution was dissolved in ethanol to concentration of 5 × 10^−5^ mol/dm^3^ (Supplementary Materials (SM), Table S1).

*Pd(II) chloride complex ions.* The proper volume of a stock solution of Pd(II) of 0.0939 mol/dm^3^ (Mennica Państwowa, Poland, purity 99.99%) was diluted in ethanol.

*Metal cations*. The sources of metal cations were the respective chlorate salts of Na(I), Ni(II), Co(II), Zn(II), Al(III), Mg(II), Li(I), and Ca(II) (Avantor Performance Materials Poland, Gliwice, Poland). Aqueous stock solutions with concentrations of 0.1 mol/dm^3^ and 0.01 mol/dm^3^ were prepared and used in the study. To obtain the desired concentration, the appropriate amount of salt was weighed and dissolved in ethanol directly in a 10 mL volumetric flask containing a fixed amount of Pd(II).

### 3.2. Methods of Analysis

*Spectrophotometry UV–Vis.* The UV–Vis spectra of the reagents were recorded using a UV–Vis spectrophotometer (Shimadzu, Kyoto, Japan) operating within the wavelength range of 190–900 nm. The instrument was equipped with a reference and thermostatic cell. All measurements were performed using 1 cm path length quartz cuvettes (Hellma, Müllheim, Germany). Spectra were collected immediately after mixing the reagents, as well as after incubation periods of 1 h, 24 h, and 7 days. The resulting data were analyzed using Origin 2021b software (OriginLab Corporation, Northampton, MA, USA).

*Fluorescence Spectroscopy.* Fluorescence spectra were measured on the spectrofluorometer FS5 (Edinburgh Instruments, Livingston, UK). Samples were analyzed in ethanol solution over the emission wavelength range of 440–800 nm, with a spectral resolution of 1 nm. The excitation wavelength was set at 423 nm. All measurements were conducted under identical instrumental settings to ensure the comparability of the results.

*Density Functional Theory (DFT) Calculation.* The optimization of the structure of the TR OO molecule was calculated by DFT using the B3LYP function and the 6–311 g basis for molecules in the anion form. The Conductor-like Polarizable Continuum Model (CPCM) was used as the solvent model to simulate ethanol. The key parameters are the ethanol dielectric constant (ε = 24.852) and the refractive index (n = 1.3611). The TD-DFT technique was used to calculate electronic spectra using the same function and database. For structures containing palladium, the LanL2DZ database was used for all calculations. The calculations were performed in the Gaussian 16 program, and the GaussView 5.0.8 program was used to visualize the data [48,49].

## 4. Conclusions

In this work, a tandem method based on UV–Vis spectrophotometry and fluorescence spectroscopy was developed for the detection of Pd(II) ions in ethanol. The combination of these two techniques allowed both precise monitoring of the complex formation process and rapid, sensitive detection of Pd(II) ions in ethanol. The optimal conditions for detection were established as a volume ratio of 3.0 mL Pd(II) to 1.0 mL TR OO and a temperature of 50 °C. Under these conditions, the most distinct color change and pronounced spectral shifts in the UV–Vis range were observed within 1 h, whereas a clear emission response was recorded after 10 min via fluorescence spectroscopy.

The performance of the proposed method was further evaluated by limit of detection (LOD) analysis, which confirmed that Pd(II) ions can be detected at concentrations as low as 1 × 10^−5^ mol/dm^3^ (1.06 ppm) using fluorescence measurements after only 10 min. The UV–Vis spectrophotometry allowed quantitative detection after 1 h, with detection possible from 1 × 10^−5^ mol/dm^3^ (1.06 ppm) at 535 nm and from 3 × 10^−5^ mol/dm^3^ (3.19 ppm) using two characteristic wavelengths (535 and 664 nm). The kinetic analysis, supported by spectrophotometric measurements, confirmed the stepwise nature of complex formation. These observations were in agreement with the results of DFT calculations, which helped to visualize the structure of the Pd(II)–TR OO complex and to explain the spectral features observed during the experiment.

The developed method allowed the detection of Pd(II) ions in the presence of selected metal cations. At higher concentrations (0.1 mol/dm^3^), reliable detection was possible in the presence of Li(I) and Na(I). At lower concentrations (0.01 mol/dm^3^), the possibility of quantitative and qualitative detection was extended to additional cations such as Al(III), Ni(II), Mg(II), Ca(II), and Co(II) after 1 h. In spectrofluorimetric analysis, fluorescence quenching by certain cations was observed, but this effect was significantly reduced at lower cation concentrations. For Zn(II), the cation with the strongest interference, the concentration had to be reduced to 0.0001 mol/dm^3^ to allow reliable detection of the Pd(II)–TR OO complex.

The proposed method is simple, fast, and cost-effective. It does not require complicated sample preparation and can be used to detect palladium in organic solvents, especially in pharmaceutical and catalytic applications. The use of a commercially available TR OO as a ligand allows easy implementation without the need for chemical modification. The method enables both qualitative and quantitative detection, with measurable signals already observable after a few minutes. Despite its merits, the method has certain limitations—mainly related to partial interference from specific metal cations at high concentrations, such as Zn(II) or Co(II). In future studies, this approach could be extended to other metal ions or applied in different solvent systems. Future research may focus on a more detailed investigation of the reaction kinetics and mechanism of complex formation between Pd(II) and azo-ligands, kinetic modeling, and extended DFT methods. Such studies could help to optimize the system further and provide deeper insight into the molecular basis of selectivity and sensitivity.

## Figures and Tables

**Table 1:** The values of wavelength (λ) and molar coefficient (ε) for the Pd(II) solution, T = 20 °C.

λ	ε
nm	M^−1^×cm^−1^
215	8765 ± 110
243	6296 ± 91
319	470 ± 20
423	264 ± 7

**Table 2:** The values of wavelength (λ) and molar coefficient (ε) for the TR OO solution, T = 20 °C.

λ	ε
nm	M^−1^×cm^−1^
270	21,034 ± 51
424	8843 ± 31

## Data Availability

The original contributions presented in this study are included in the article/Supplementary Materials. Further inquiries can be directed to the corresponding author(s).

## Supplementary Materials

The supporting information can be downloaded at https://www.mdpi.com/article/10.3390/ijms26125613/s1.
