# Supplementary material for: High-Sensitivity Sensor for Palladium Detection in Organic Solvent
Source: Int J Mol Sci. 2025 Jun 11;26(12):5613. doi: 10.3390/ijms26125613 (PMC12193551; doi:10.3390/ijms26125613)
Supplement: Supplementary file 1 [file ijms-26-05613-s001.zip › ijms-3652213-supplementary.pdf]

Supplementary Materials

High-sensitivity sensor for palladium detection in organic solvent

Adrianna Pach [1], Agnieszka Podborska [2] and Magdalena Luty-Błocho [1,*]

[1] AGH University of Krakow, Faculty of Non-Ferrous Metals, al. A. Mickiewicza 30, 30-059 Krakow, Poland.; apach@agh.edu.pl
2 AGH University of Krakow, Academic Centre for Materials and Nanotechnology, al. A. Mickiewicza 30, 30-059 Krakow, Poland; podborsk@agh.edu.pl
* Correspondence: mlb@agh.edu.pl

1. Experimental conditions

Table S1. Experimental conditions.

Initial concentration of reagents		Volumetric ratio of TR OO to Pd(II) ions	T
$C_{0,TR\ OO}$, mol/dm³	$C_{0,Pd\ (II)}$, mol/dm³	$V_{TR\ OO} : V_{Pd\ (II)}$, mL/mL	°C
The stoichiometry			
5 × 10⁻⁵	5 × 10⁻⁵	0.5 : 3.5 (A)	20
		1.0 : 3.0 (B)	50
		1.5 : 2.5 (C)	
		2.0 : 2.0 (D)	
		2.5 : 1.5 (E)	
		3.0 : 1.0 (F)	
		3.5 : 0.5 (G)	
Determine the limit detection (LOD) of the Pd (II) ions			
5 × 10⁻⁶	5 × 10⁻⁶	1.0 : 3.0	50
1 × 10⁻⁵	1 × 10⁻⁵		
3 × 10⁻⁵	3 × 10⁻⁵		
5 × 10⁻⁵	5 × 10⁻⁵		
The influence of cations (Li^+, Na^+, Al^{3+}, Ni^{2+}, Mg^{2+}, Ca^{2+}, Co^{2+}, Zn^{2+}) derived from dissociation of ClO_4^- salts. The concentration of anions in the sample was set as 0.1 and 0.01 mol/dm³ (after mixing the reagents)			
5 × 10⁻⁵	5 × 10⁻⁵	1.0 : 3.0	50

2. Spectra of reagents

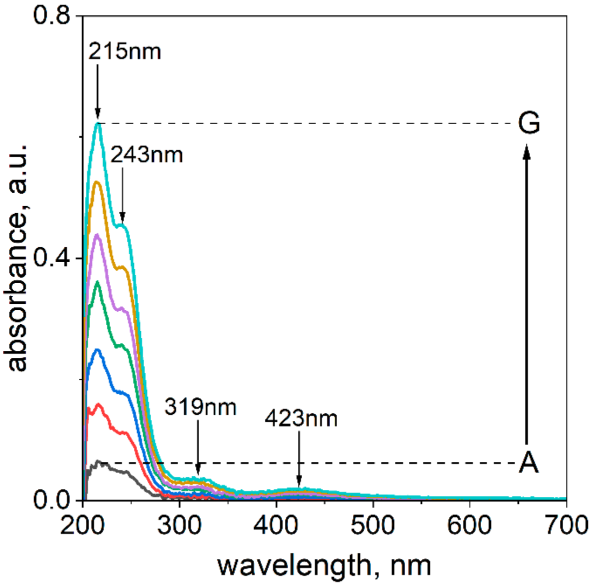

(a)

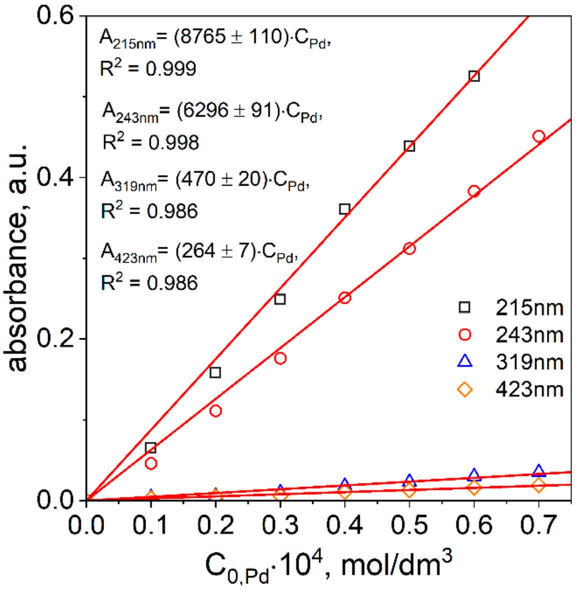

(b)

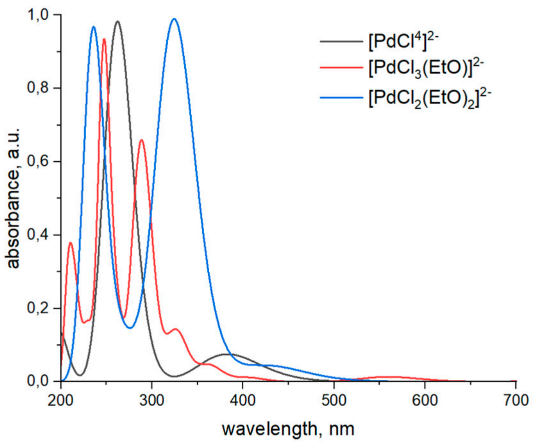

(c)

Figure S1. The UV–Vis spectra of Pd(II) ions in ethanol at different initial concentrations: A—6.25 × 10^{-6} mol/dm^3; B—1.25 × 10^{-5} mol/dm^3; C—1.87 × 10^{-5} mol/dm^3; D—2.5 × 10^{-5} mol/dm^3; E—3.125 × 10^{-5} mol/dm^3; F—5 × 10^{-5} mol/dm^3; G—4.38 × 10^{-5} mol/dm^3 (a), dependency of absorbance vs. initial concentrations of Pd(II) ions at different wavelengths: 215, 243, 319, and 423 nm (b), TD-DFT calculated spectra for different forms of Pd(II) ions in ethanol (c). Conditions: T = 20 °C, path length 1 cm.

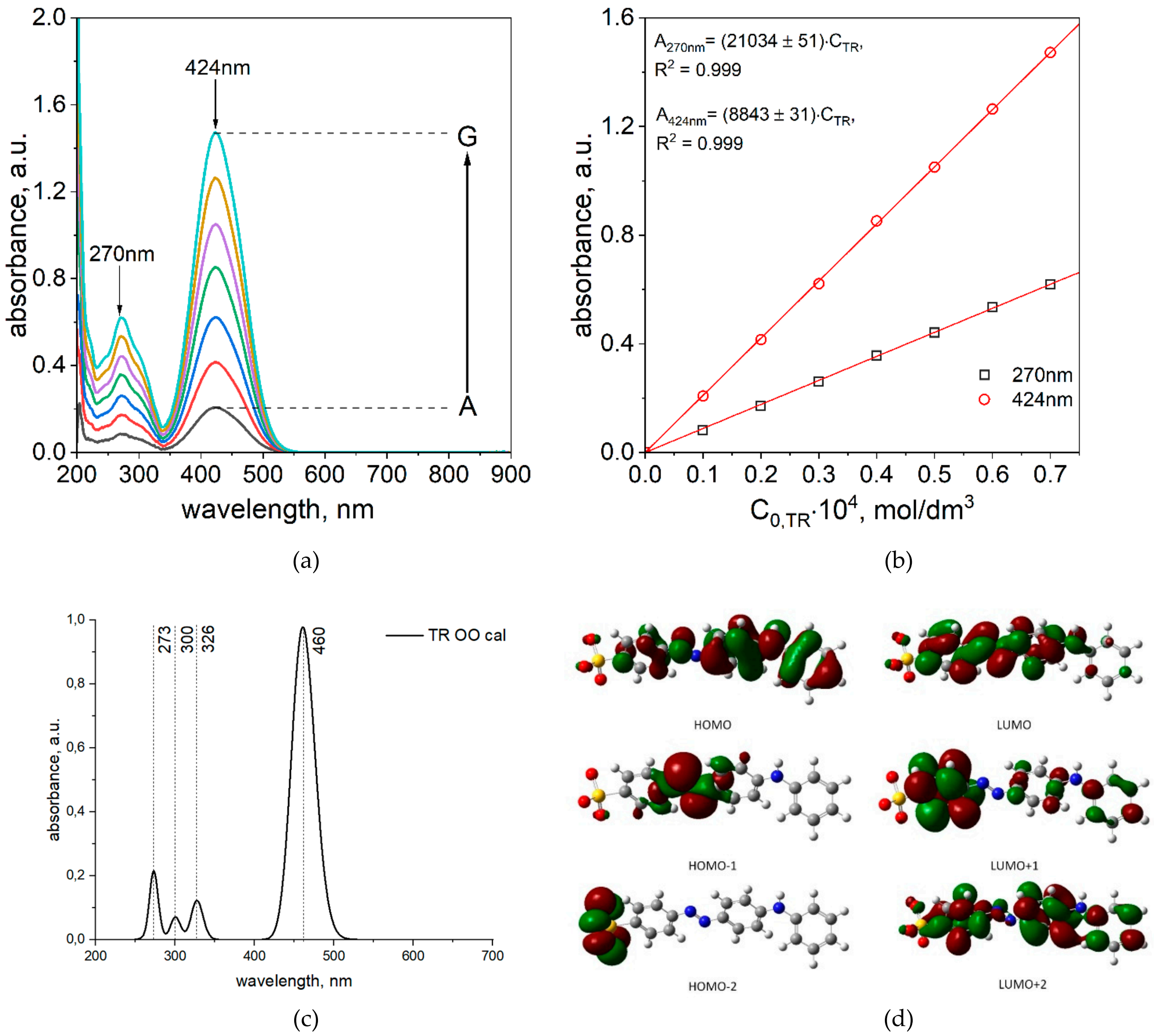

Figure S2. The UV–Vis spectra of tropaeolin OO (TR OO) in ethanol at different initial concentrations: A—6.25 × 10^{-6} mol/dm^3; B—1.25 × 10^{-5} mol/dm^3; C—1.87 × 10^{-5} mol/dm^3; D—2.5 × 10^{-5} mol/dm^3; E—3.125 × 10^{-5} mol/dm^3; F—3.75 × 10^{-5} mol/dm^3; G—4.38 × 10^{-5} mol/dm^3 (a), dependency of absorbance vs. initial concentrations of TR OO at different wavelengths: 270 and 424 nm, (b) TD-DFT calculated spectrum for TR OO (c), orbitals calculated for the TR OO molecule in ethanol (d). Conditions: T = 20 °C, path length 1 cm.

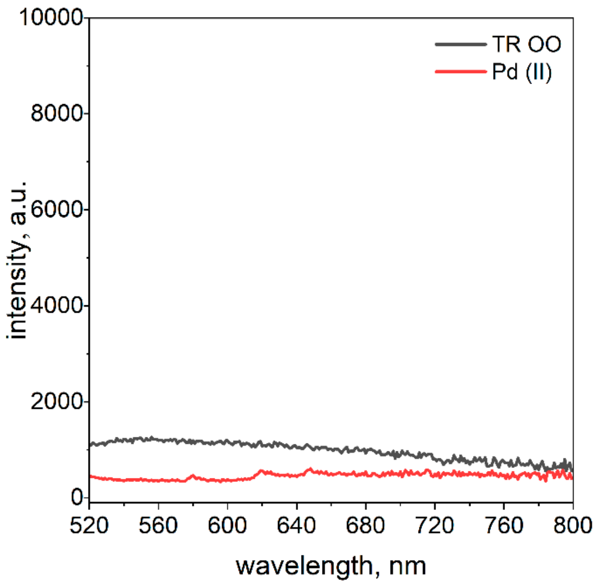

Figure S3. The fluorescence emission spectra of solutions containing TR OO (black) and Pd(II) ions (red). Conditions: the value of concentration before reagent mixing, $C_{0,TR\ OO\ =\ Pd(II)} = 5 \times 10^{-5}$ mol/dm^3; T = 20 °C; path length, 1 cm.

3. Optimalization and characterization of Pd (II) – TR OO complex formation

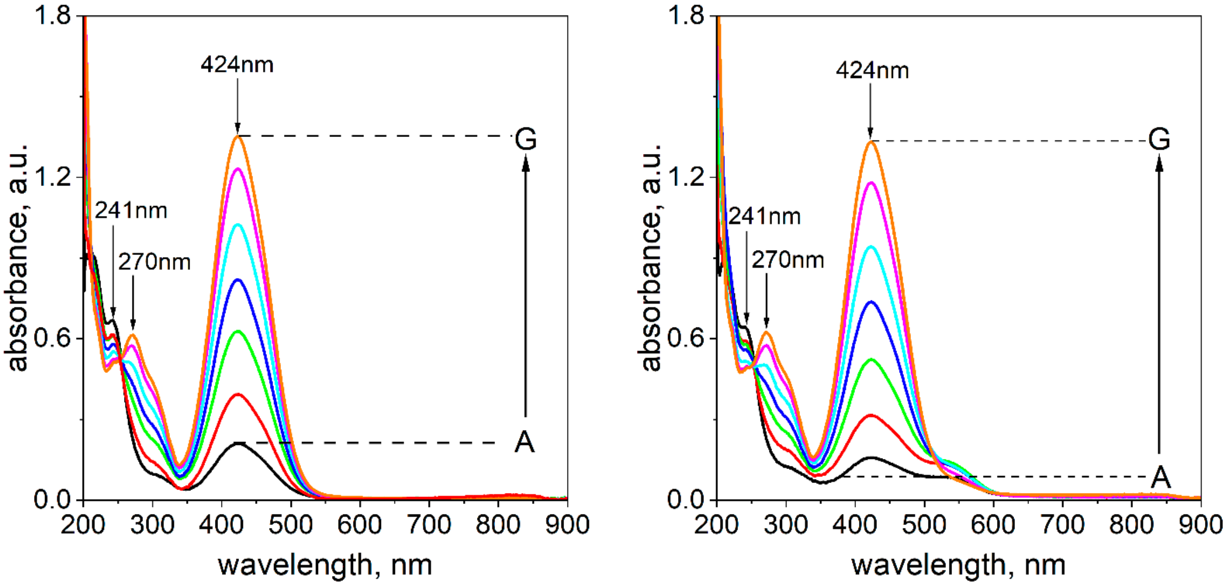

(a) (b)

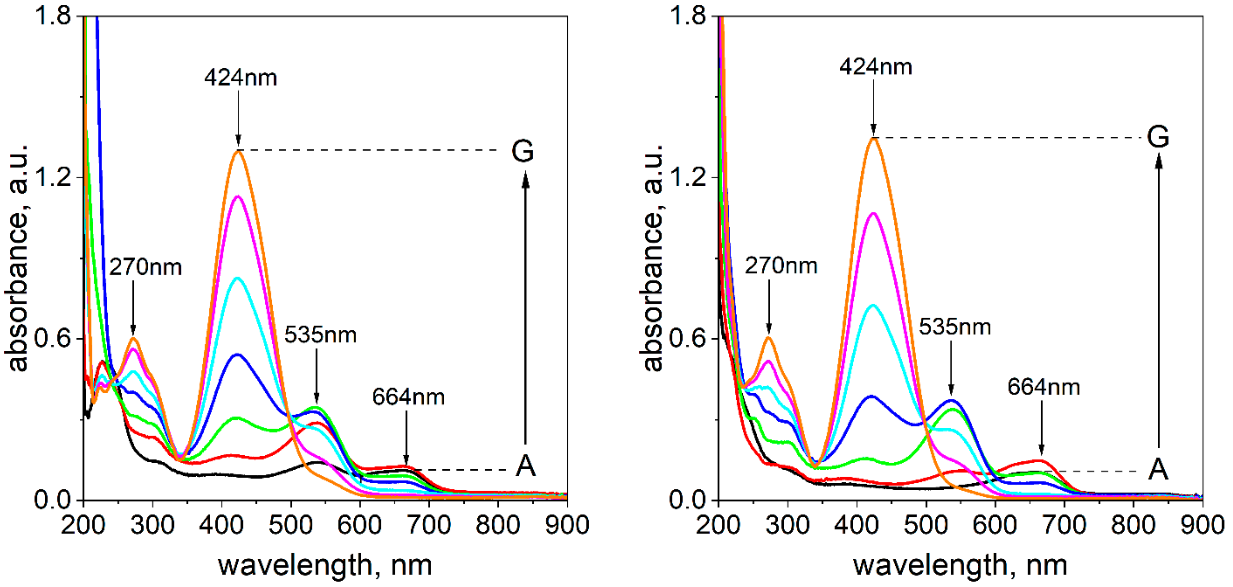

(c) (d)

Figure S4. The UV–Vis spectra of solutions obtained after the mixing of TR OO with Pd(II) ions at different volumetric ratios of Pd(II) to TR OO, A—3.5:0.5; B—3.0:1.0; C—2.5:1.5; D—2.0:2.0, E—1.5:2.5; F—1.0:3.0; G—0.5:3.5 (mL/mL), after 5 min (a), 1 h (b), 24 h (c), and 7 days (d). Conditions: the value of concentration before reagent mixing, $C_{0,TR\ OO} = C_{0,Pd(II)} = 5 \times 10^{-5}$ mol/dm^3; T = 20 °C; path length, 1 cm.

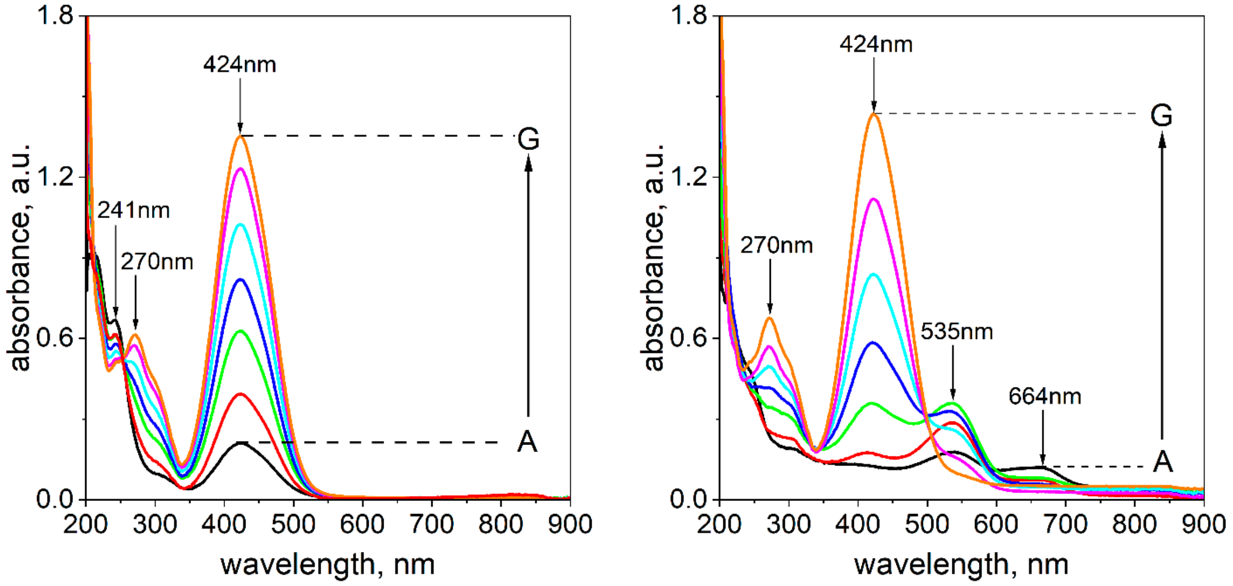

(a) (b)

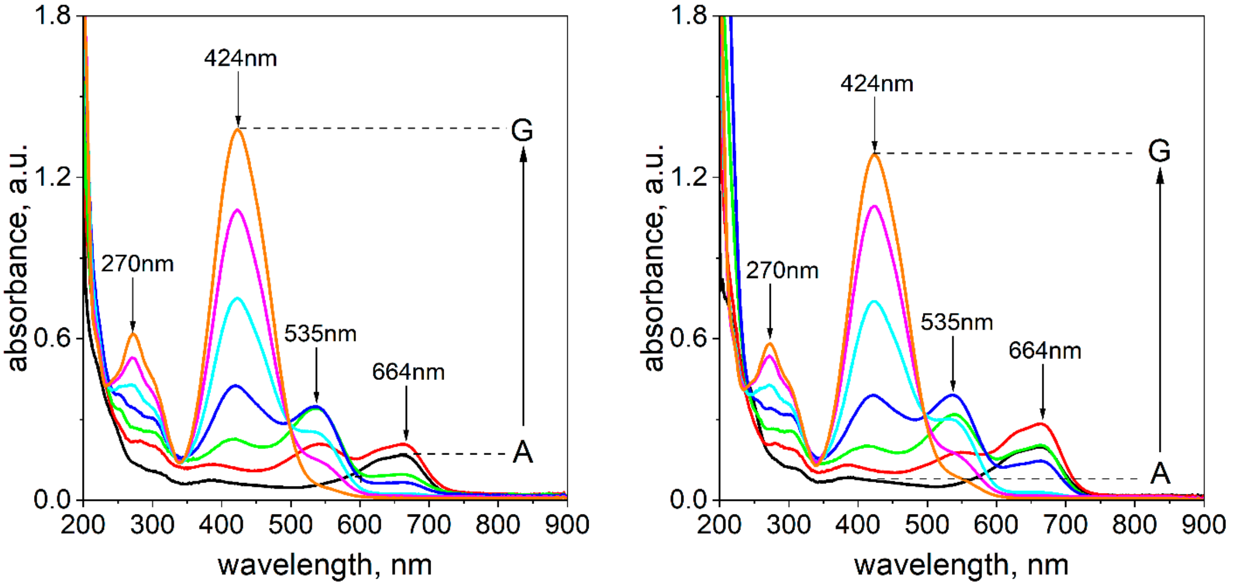

(c) (d)

Figure S5. The UV–Vis spectra of solutions obtained after the mixing of TR OO with Pd(II) ions at different volumetric ratios of Pd(II) to TR OO, A—3.5:0.5; B—3.0:1.0; C—2.5:1.5; D—2.0:2.0, E—1.5:2.5; F—1.0:3.0; G—0.5:3.5 (mL/mL), after 5 min (a), 1 h (b), 24 h (c), and 7 days (d). Conditions: the value of concentration before reagent mixing, $C_{0,TR\ OO}$= $C_{0,Pd(II)}$ = 5 × 10^{-5} mol/dm^3; T = 50 °C; path length, 1 cm.

4. Job's plot

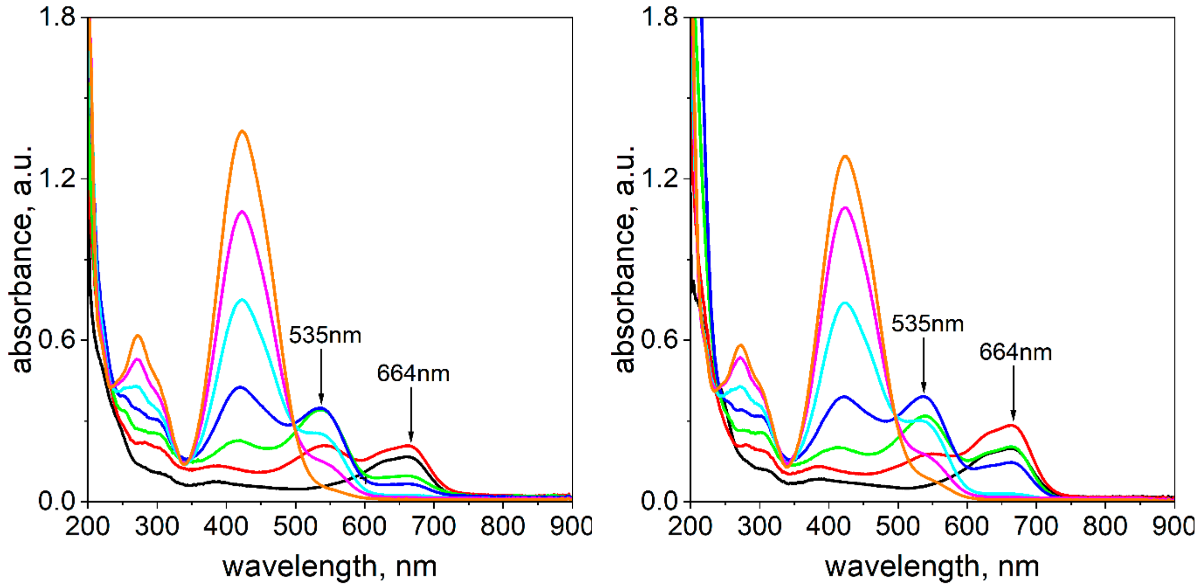

(a) (b)

Figure S6. The UV–Vis spectra of solutions obtained after mixing of Pd(II) ions with TR OO in ethanol as a solvent, at different volumetric ratios after 24 h (a) and 7 days (b). Conditions: the value of concentration before reagent mixing, $C_{0,TR\ OO}$ = $C_{0,Pd(II)}$ = 5 × 10^{-5} mol/dm^3; T = 50 °C.

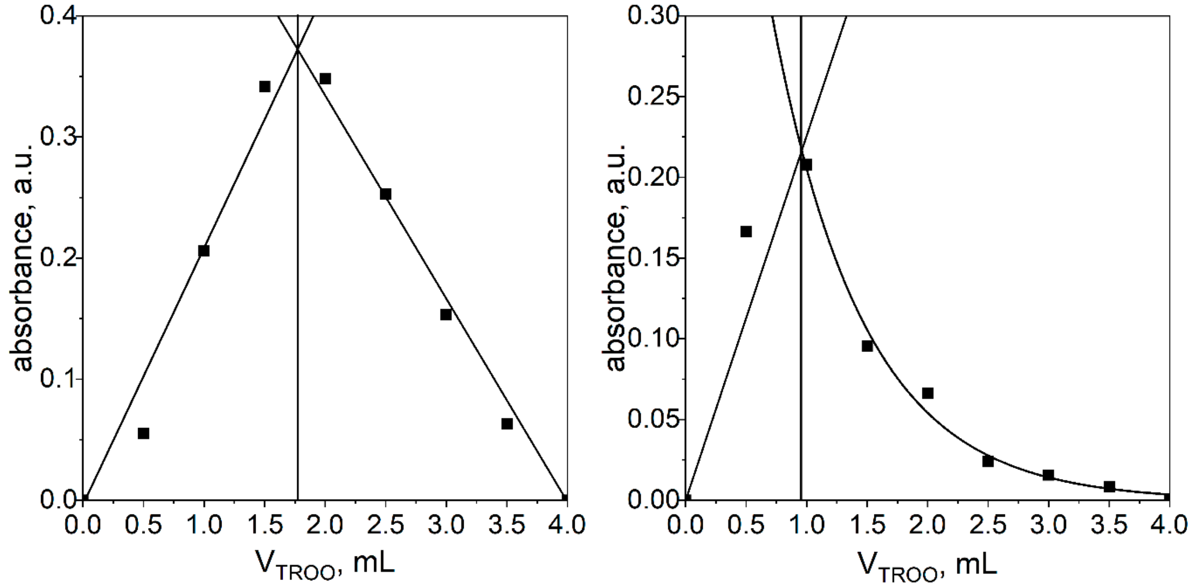

(a) (b)

Figure S7. The dependency of absorbance vs. ratio of TR OO to Pd(II) volumes, registered at 535 nm (a) and at 664 nm (b) after 24 h. Conditions: $C_{0,TR\ OO} = C_{0,Pd(II)} = 5 \times 10^{-5}$ mol/dm^3, total volume of reagent = 4.0 mL, T = 50 °C.

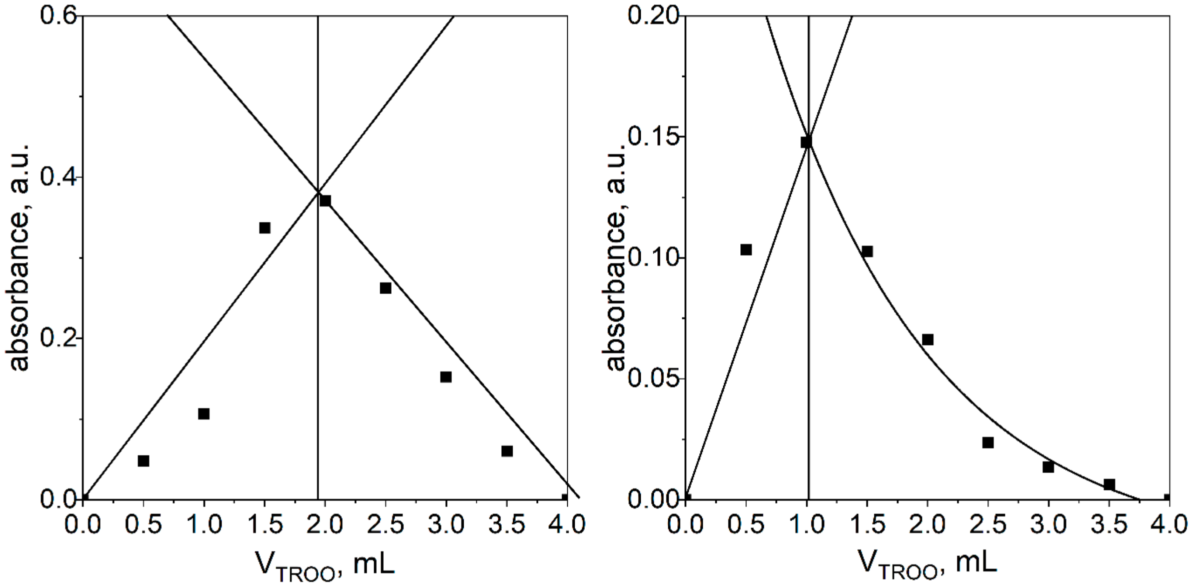

(a) (b)

Figure S8. The dependency of absorbance vs. ratio of TR OO to Pd(II) volumes, registered at 535 nm (a) and at 664 nm (b) after 7 days. Conditions: $C_{0,TR\ OO} = C_{0,Pd(II)} = 5 \times 10^{-5}$ mol/dm^3, total volume of reagent = 4.0 mL, T = 50 °C.

5. Kinetics and Mechanism of Pd (II)-TROO Complex Formation

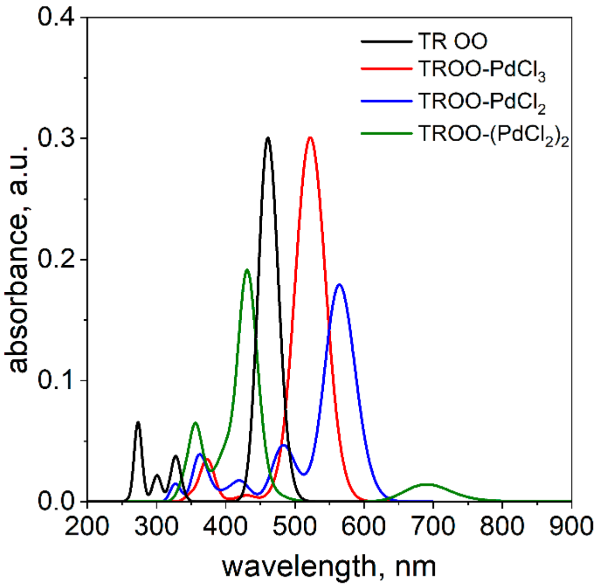

Figure S9. TD-DFT calculated spectra for all Pd(II)–TR OO complexes.

6. Determining the limit of detection of the Pd (II) ions for the Pd(II)-TR OO complex

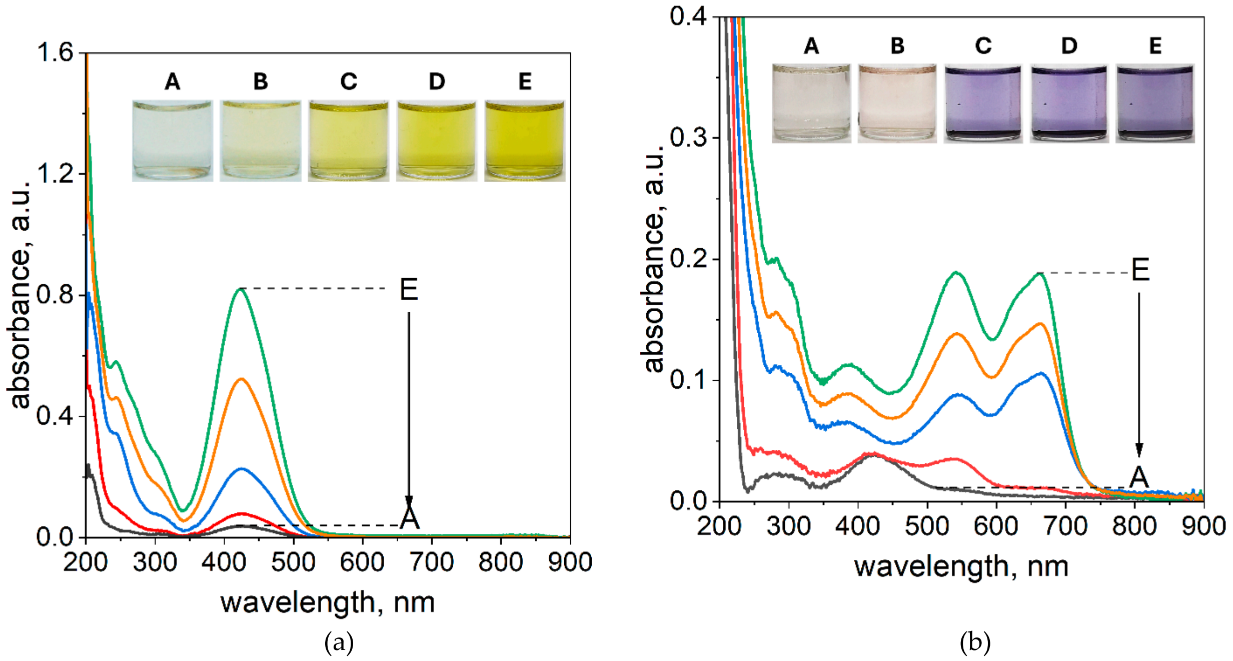

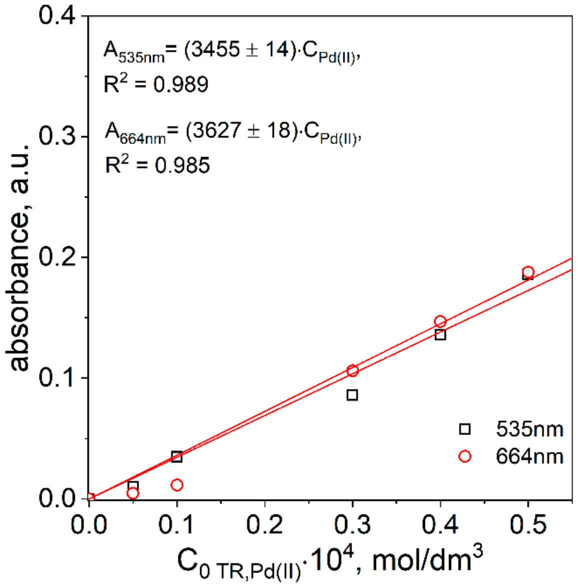

(c)

Figure S10. Spectra of solutions containing a mixture of TR and Pd(II) ions after 5 min (a) and 24 h (b); dependency of absorbance vs. initial concentration of Pd(II) ion after 1 h (c). Conditions: $C_{0,TR\ OO/Pd(II)}$ = A—5 × 10^{-6} mol/dm^3; B—1 × 10^{-5} mol/dm^3; C—3 × 10^{-5} mol/dm^3; D—4 × 10^{-5} mol/dm^3, E—4 × 10^{-5} mol/dm^3. T = 50 °C, path length 1 cm.

7. The influence of metal cations

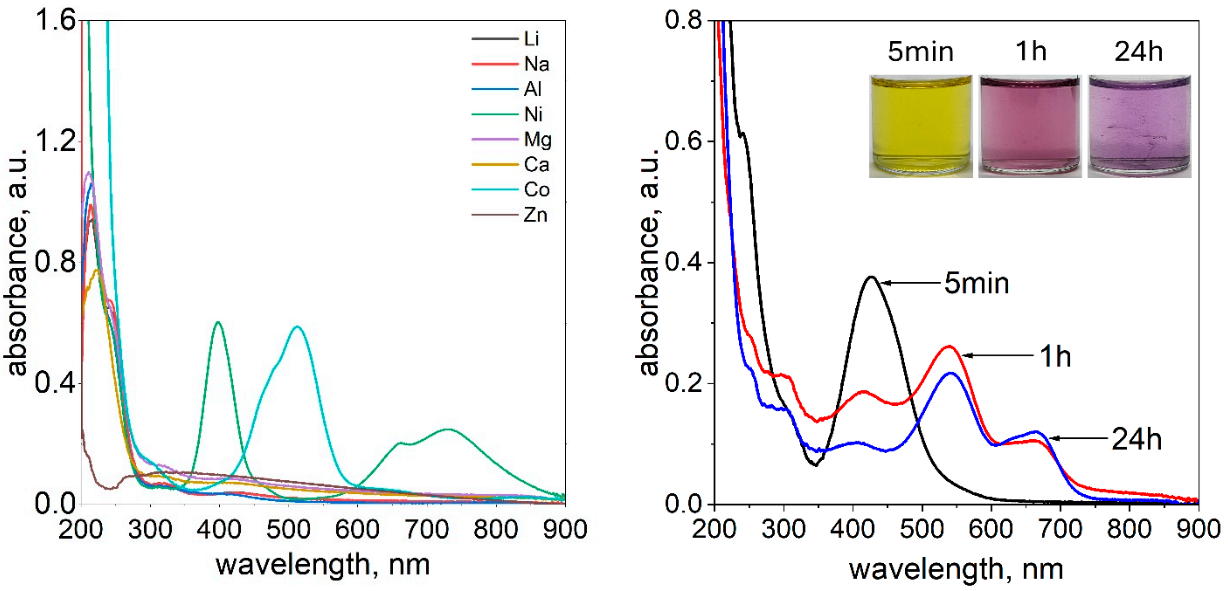

(a)

(b)

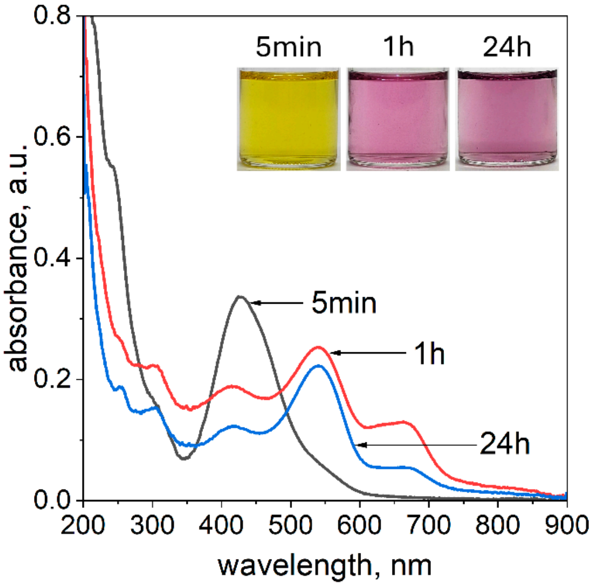
absorbance, a.u.
wavelength, nm
0.0
0.2
0.4
0.6
0.8
200
300
400
500
600
700
800
900
5min
1h
24h
5min
1h
24h

(c)

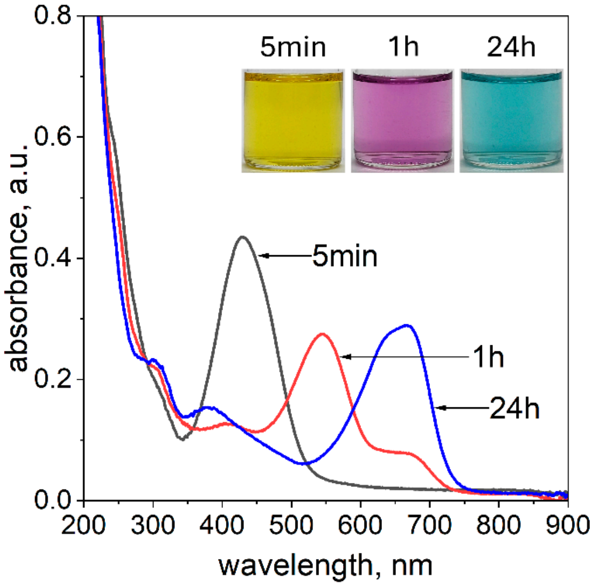
absorbance, a.u.
wavelength, nm
0.0
0.2
0.4
0.6
0.8
200
300
400
500
600
700
800
900
5min
1h
24h
5min
1h
24h

(d)

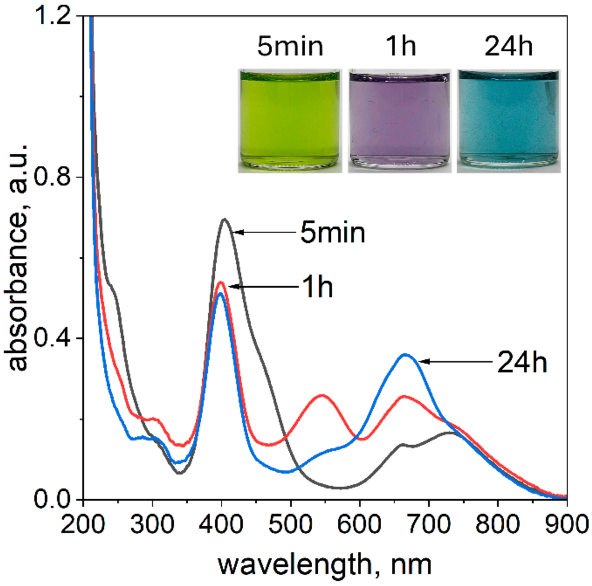
absorbance, a.u.
wavelength, nm
0.0
0.4
0.8
1.2
200
300
400
500
600
700
800
900
5min
1h
24h
5min
1h
24h

(e)

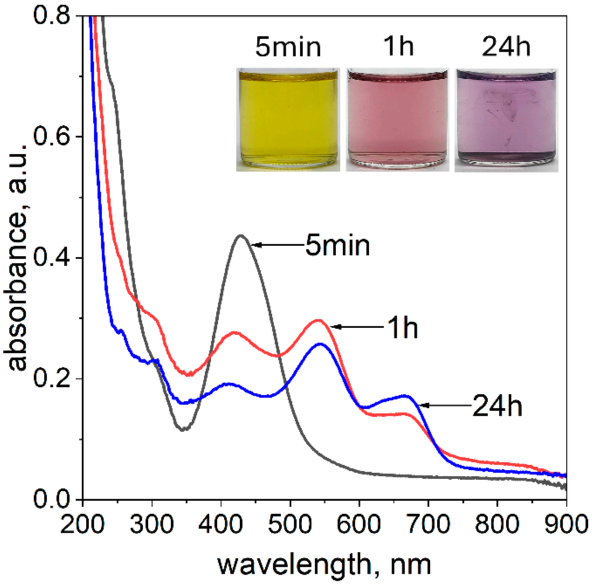
absorbance, a.u.
wavelength, nm
0.0
0.2
0.4
0.6
0.8
200
300
400
500
600
700
800
900
5min
1h
24h
5min
1h
24h

(f)

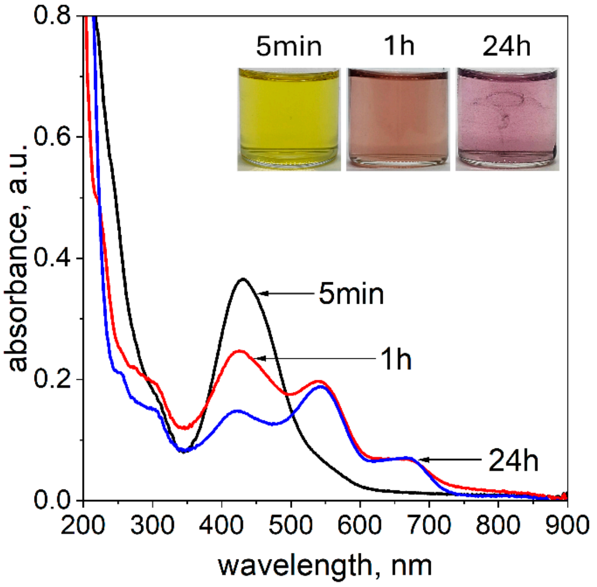

(g)

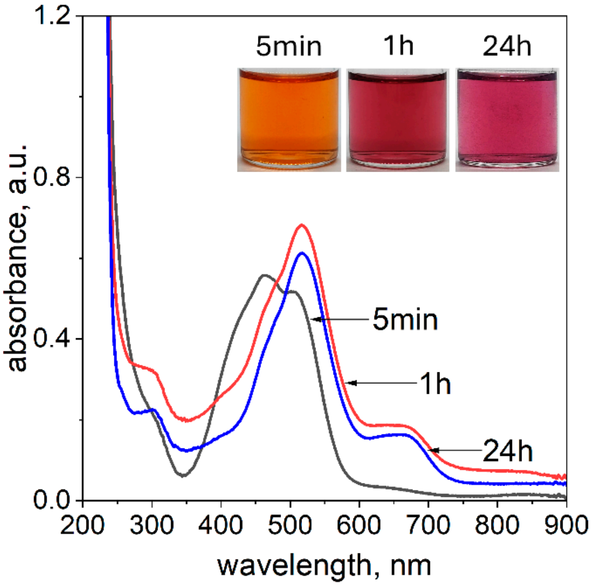

(h)

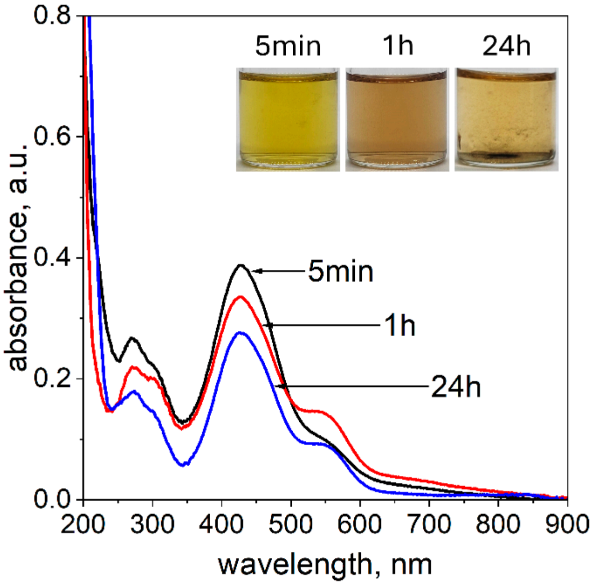

(i)

Figure S11. The evolution of UV–Vis spectra solutions containing Pd(II) with metal cations (a), Pd(II), TR OO, and other metal cations—Li^{+} (b), Na^{+} (c), Al^{3+} (d), Ni^{2+} (e), Mg^{2+} (f), Ca^{+}(g), Co^{2+} (h), and Zn^{2+} (i)—after 5 min, 1 h, and 24 h in ethanol. Conditions: $C_{0,TR\ OO}$ = 1.25 × 10^{-5} mol/dm³, $C_{0,Pd(II)}$= 3.75 × 10^{-5} mol/dm³, $C_{0,anions}$ = 0.0625 mol/dm³, volumetric ratio mixing of Pd(II) ions and TR OO = 3.0 mL: 1.0 mL, T = 50 °C.

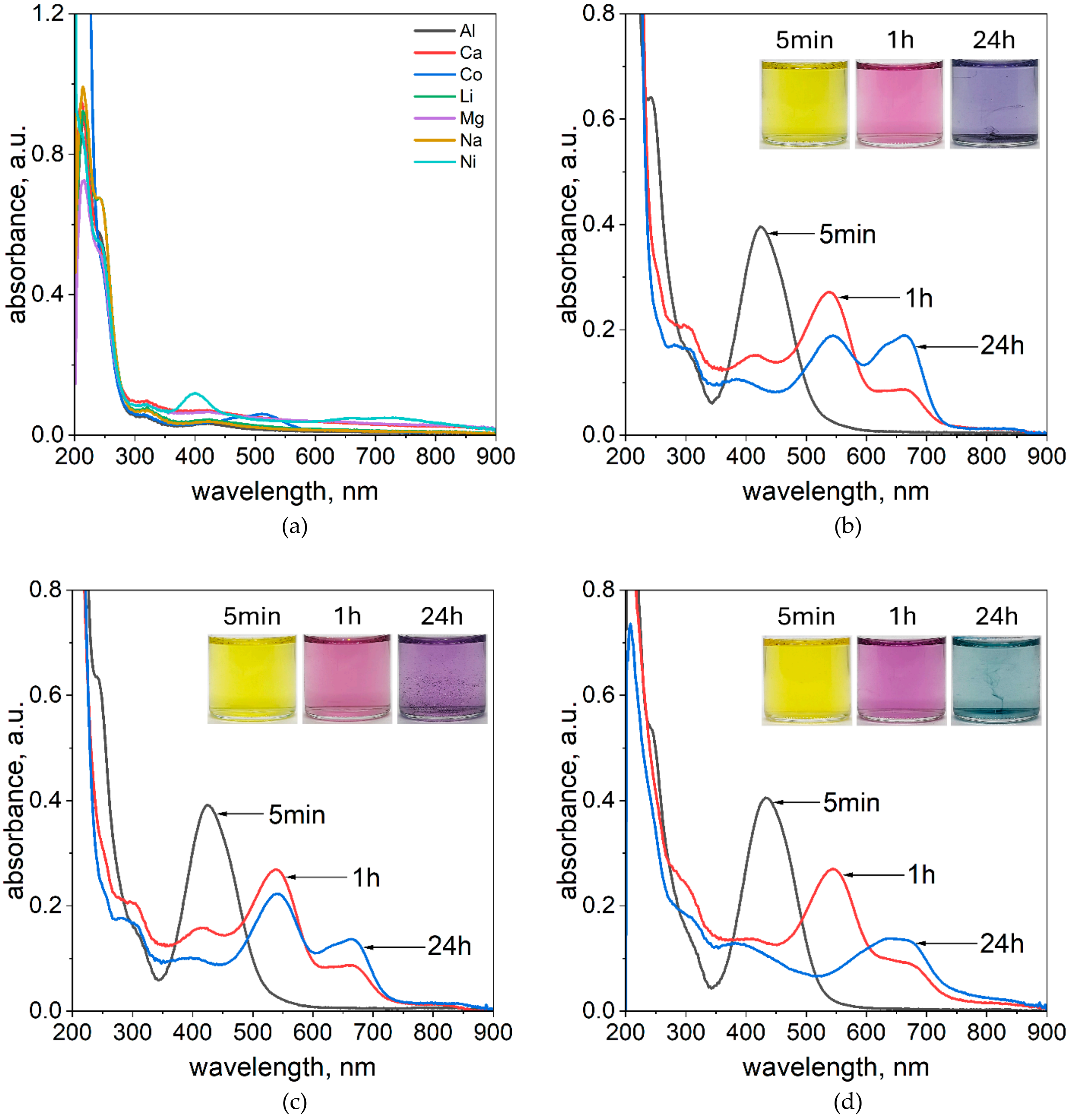

absorbance, a.u.
wavelength, nm
Al
Ca
Co
Li
Mg
Na
Ni
(a)
5min
1h
24h
(b)
(c)
(d)

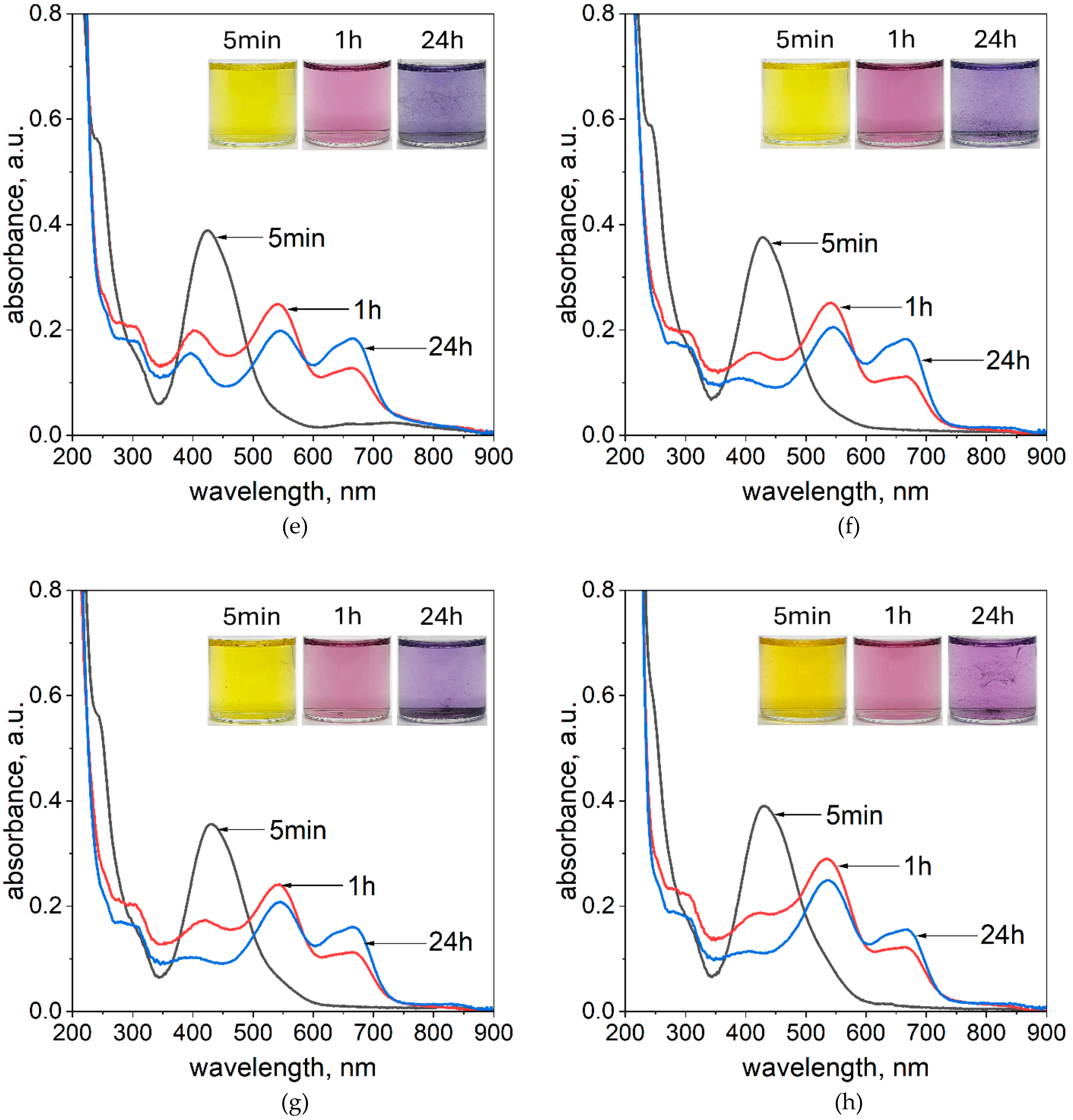

Figure S12. The evolution of UV–Vis spectra of solutions containing Pd(II) with metal cations (a), Pd(II), TR OO, and other metal cations—Li^{+} (b), Na^{+} (c), Al^{3+} (d), Ni^{2+} (e), Mg^{2+} (f), Ca^{+} (g), and Co^{2+} (h)—after 5 min, 1 h, and 24 h in ethanol. Conditions: $C_{0,TR\ OO}$ = 1.25 × 10^{-5} mol/dm³, $C_{0,Pd(II)}$= 3.75 × 10^{-5} mol/dm³, $C_{0,anions}$ = 0.00625 mol/dm³, volumetric ratio mixing of Pd(II) ions and TR OO = 3.0 mL: 1.0 mL, T = 50 °C.

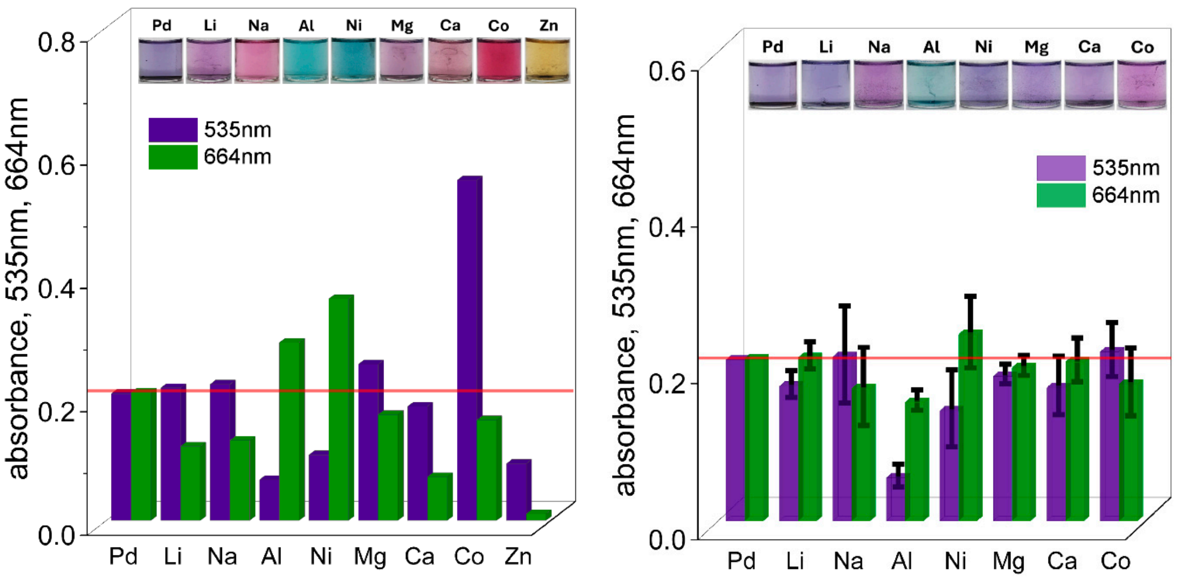

(a) (b)

Figure S13. The value of absorbance at wavelength 535 nm and 664 nm for solutions containing Pd(II) ions with TR OO and Pd(II)–TR OO with metal cations—Li^{+}, Na^{+}, Al^{3+}, Ni^{2+}, Mg^{2+}, Ca^{2+}, Co^{2+}, and Zn^{2+}—at concentrations of 0.1 mol/dm³ (a) and 0.01 mol/dm³ (b) after 24 h in ethanol. Conditions: $C_{0,TR\ OO} = 1.25 \times 10^{-5}$ mol/dm³, $C_{0,Pd(II)} = 3.75 \times 10^{-5}$ mol/dm³, volumetric ratio mixing of Pd(II) ions and TR OO = 3.0 mL: 1.0 mL, T = 50 °C.

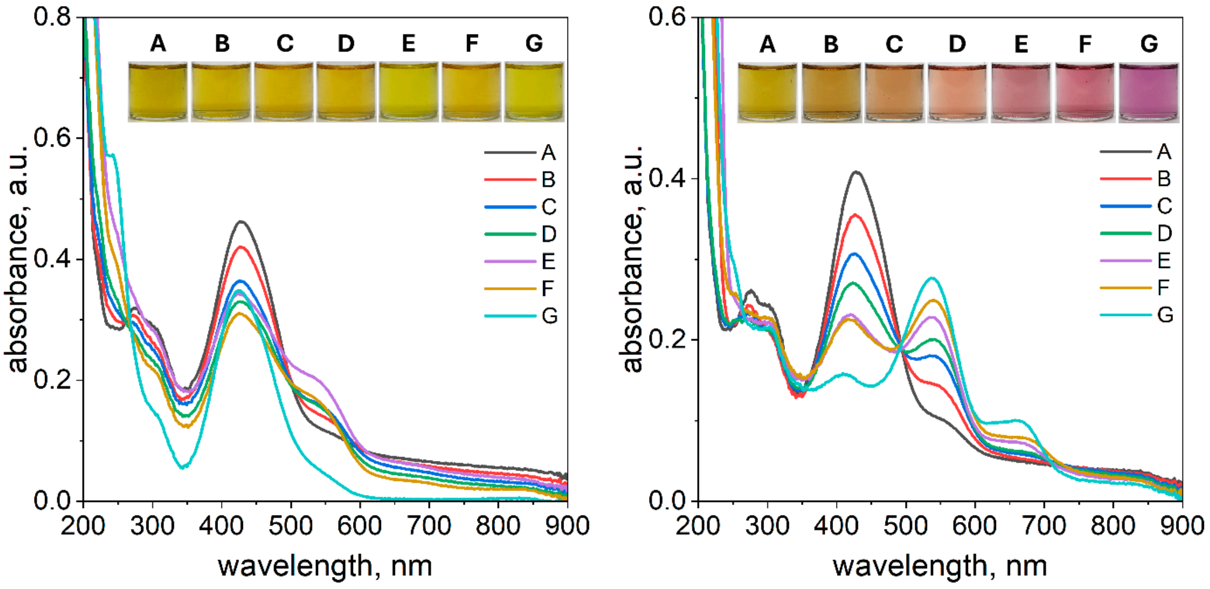

(a) (b)

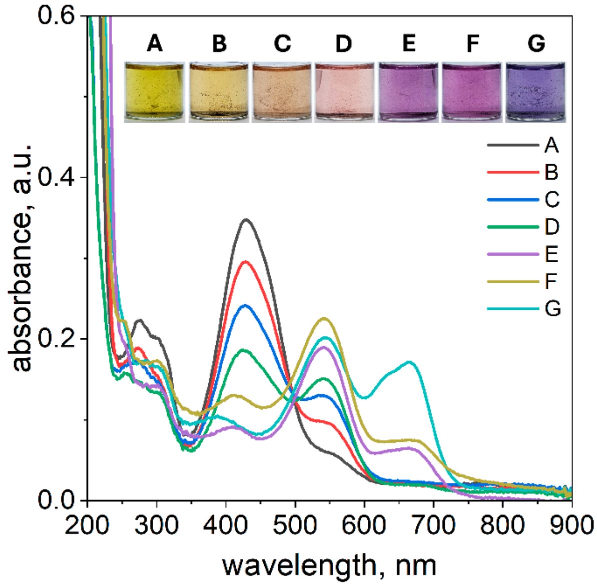

(c)

Figure S14. The evolution of UV–Vis spectra of solutions containing Pd(II), TR OO, and different concentrations of Zn^{2+}—A: 1 × 10^{-1} mol/dm^3; B: 5 × 10^{-2} mol/dm^3; C: 1 × 10^{-2} mol/dm^3; D: 5 × 10^{-3} mol/dm^3; E: 1 × 10^{-3} mol/dm^3; F: 5 × 10^{-4} mol/dm^3; G: 1 × 10^{-4} mol/dm^3—after 5 min (a), 1 h (b), and 24 h (c) in ethanol. Conditions: $C_{0,TR\ OO}$ = 1.25 × 10^{-5} mol/dm^3, $C_{0,Pd(II)}$ = 3.75 × 10^{-5} mol/dm^3, volumetric ratio mixing of Pd(II) ions and TR OO = 3.0 mL: 1.0 mL, T = 50 °C.

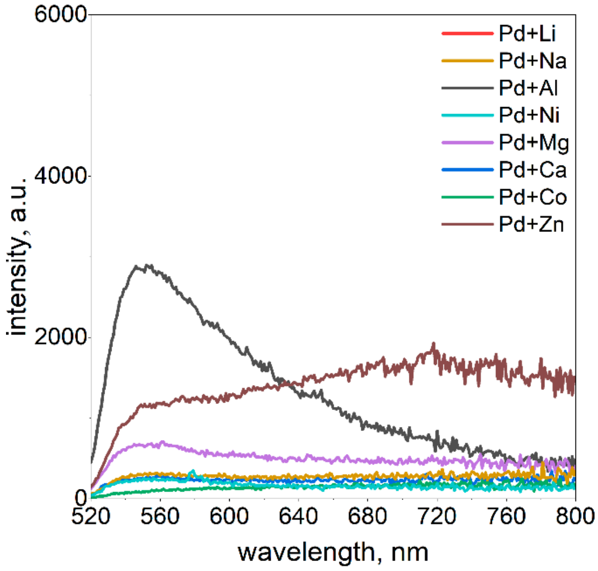

(a)

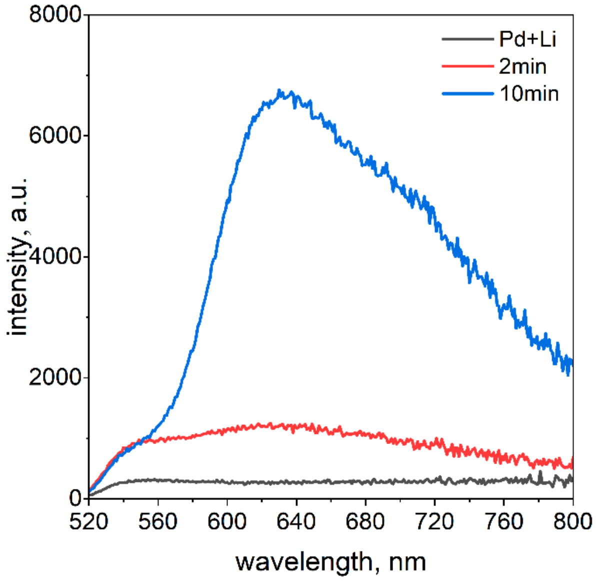

(b)

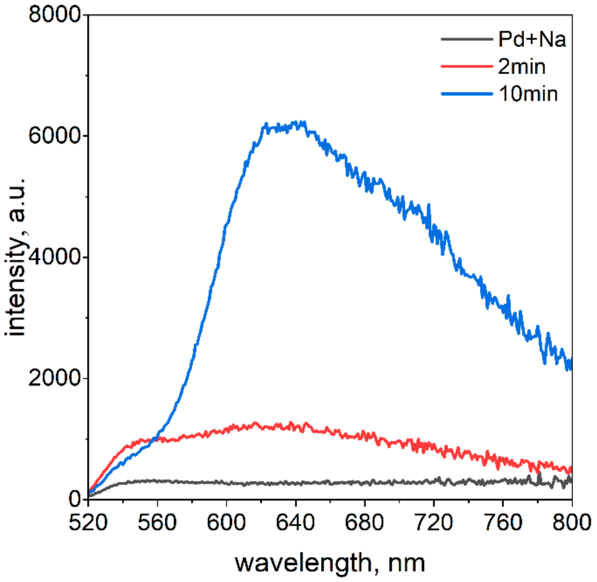
Pd+Na
2min
10min
intensity, a.u.
wavelength, nm
0
2000
4000
6000
8000
520
560
600
640
680
720
760
800
(c)

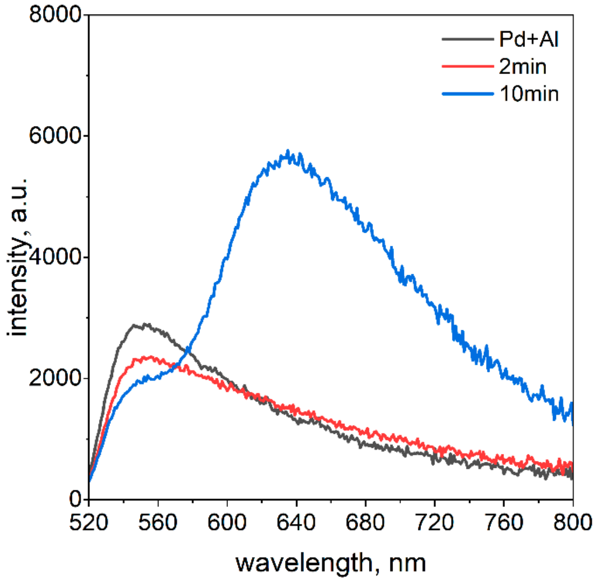
Pd+Al
2min
10min
intensity, a.u.
wavelength, nm
0
2000
4000
6000
8000
520
560
600
640
680
720
760
800
(d)

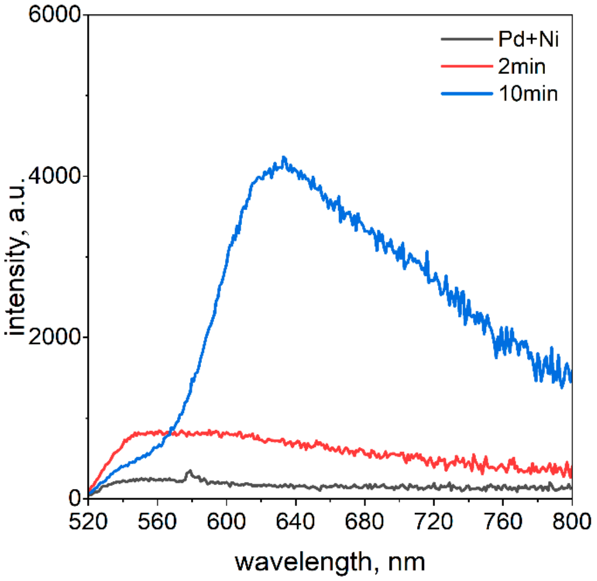
Pd+Ni
2min
10min
intensity, a.u.
wavelength, nm
0
2000
4000
6000
520
560
600
640
680
720
760
800
(e)

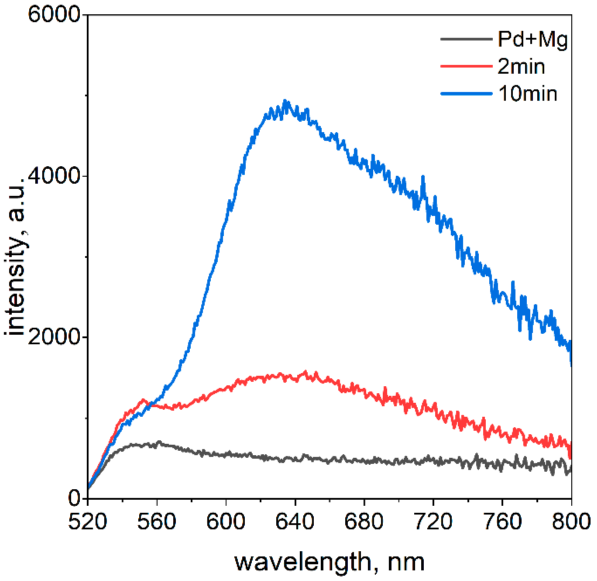
Pd+Mg
2min
10min
intensity, a.u.
wavelength, nm
0
2000
4000
6000
520
560
600
640
680
720
760
800
(f)

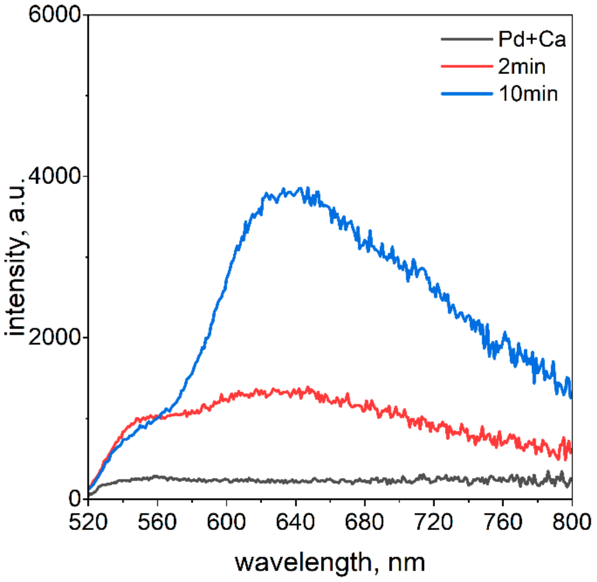

(g)

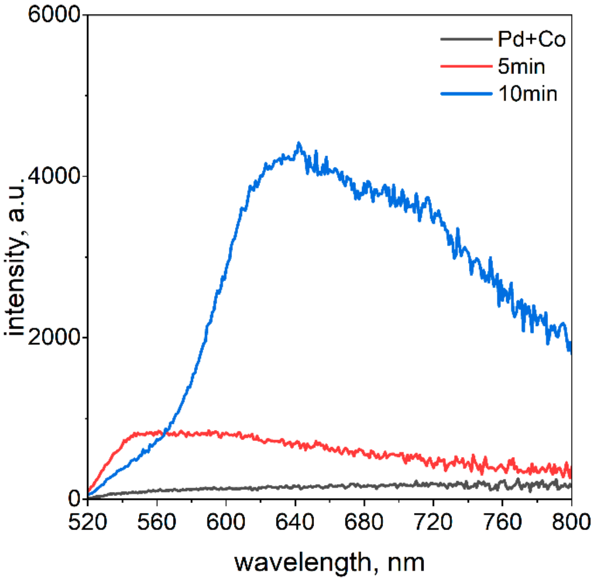

(h)

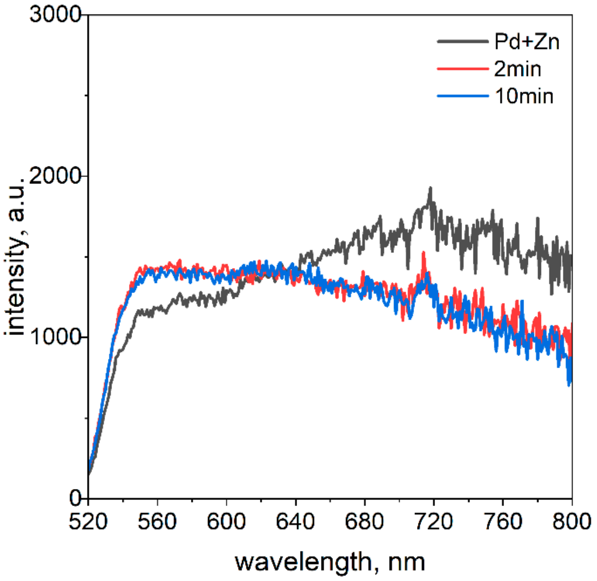

(i)

Figure S15. The evolution of fluorescence spectra of solutions containing Pd(II) with metal cations without of TR OO (a), Pd(II), TR OO, and other metal cations—Li^+ (b), Na^+ (c), Al^{3+} (d), Ni^{2+} (e), Mg^{2+} (f), Ca^{2+} (g), Co^{2+} (h), and Zn^{2+} (i)—at 2 min and 10 min after mixing in ethanol. Conditions: $C_{0,TR\ OO}$= 1.25 × 10^{-5} mol/dm^3, $C_{0,Pd(II)}$= 3.75 × 10^{-5} mol/dm^3, $C_{0,anions}$ = 0.0625 mol/dm^3, volumetric ratio mixing of Pd(II) ions and TR OO = 3.0 mL: 1.0 mL, T = 50 °C.

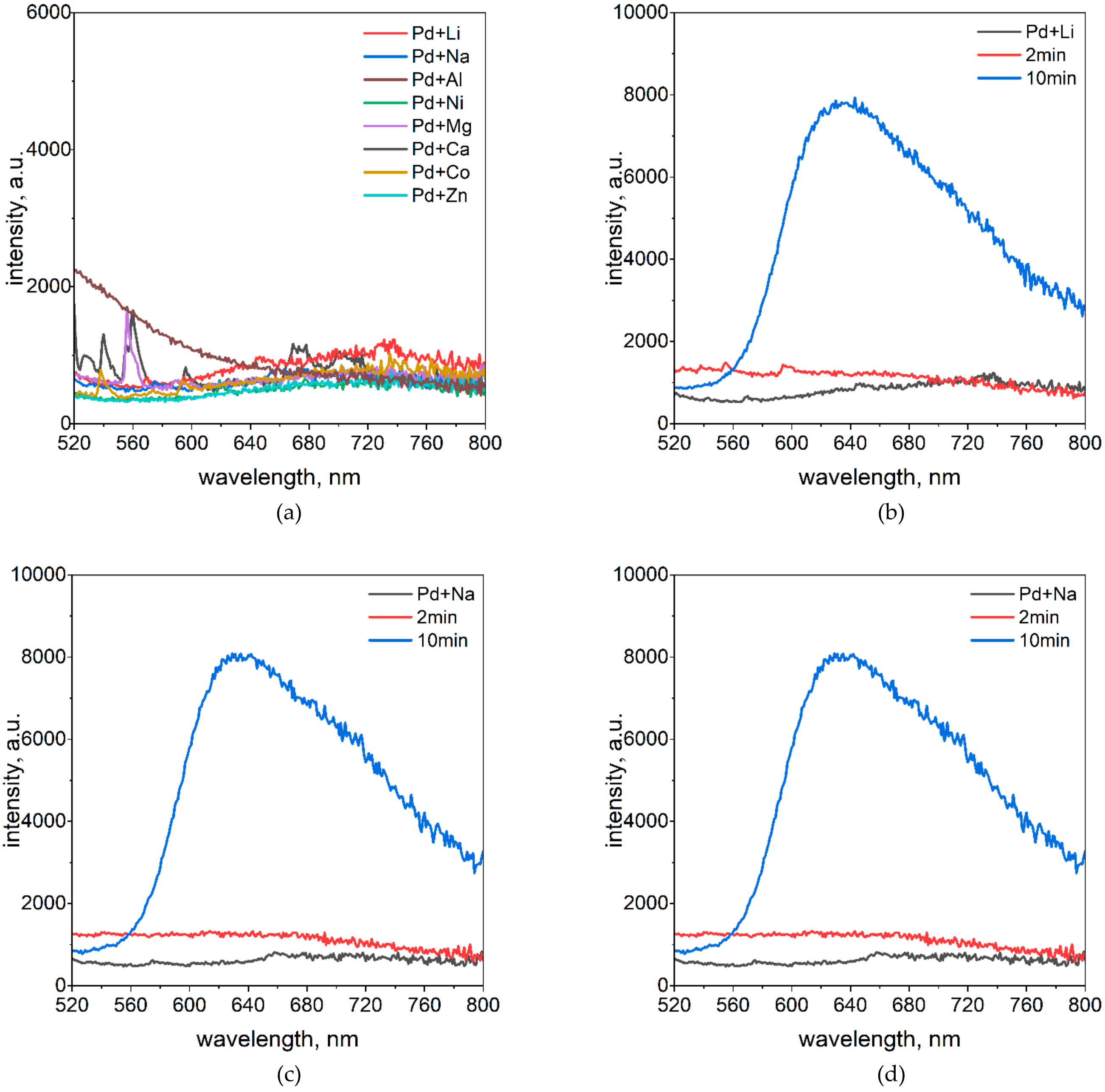

Pd+Li
Pd+Na
Pd+Al
Pd+Ni
Pd+Mg
Pd+Ca
Pd+Co
Pd+Zn
intensity, a.u.
wavelength, nm
(a)
Pd+Li
2min
10min
intensity, a.u.
wavelength, nm
(b)
Pd+Na
2min
10min
intensity, a.u.
wavelength, nm
(c)
Pd+Na
2min
10min
intensity, a.u.
wavelength, nm
(d)

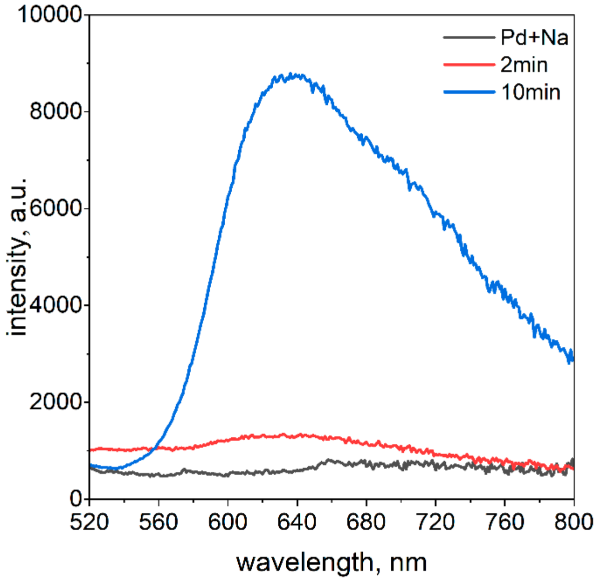
Pd+Na
2min
10min
intensity, a.u.
0
2000
4000
6000
8000
10000
520
560
600
640
680
720
760
800
wavelength, nm
(e)

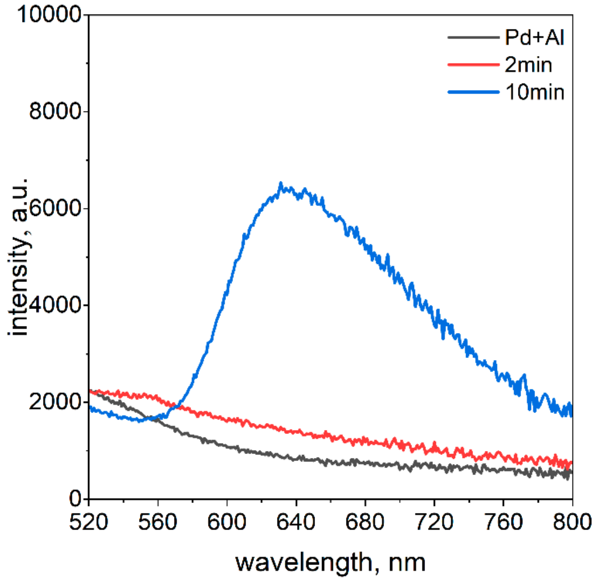
Pd+Al
2min
10min
intensity, a.u.
0
2000
4000
6000
8000
10000
520
560
600
640
680
720
760
800
wavelength, nm
(f)

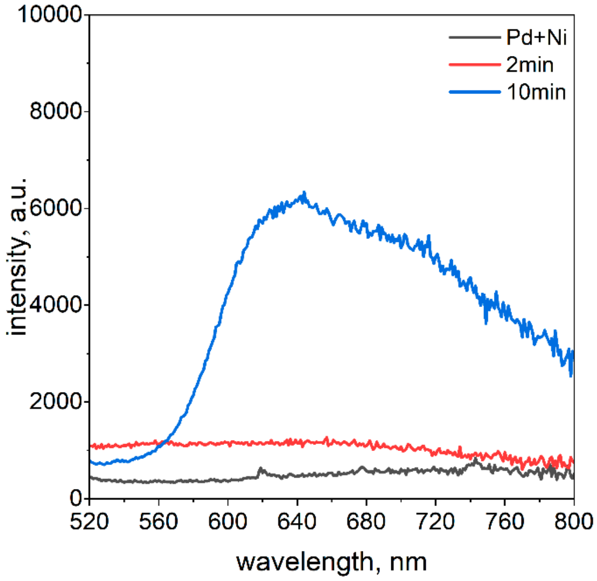
Pd+Ni
2min
10min
intensity, a.u.
0
2000
4000
6000
8000
10000
520
560
600
640
680
720
760
800
wavelength, nm
(g)

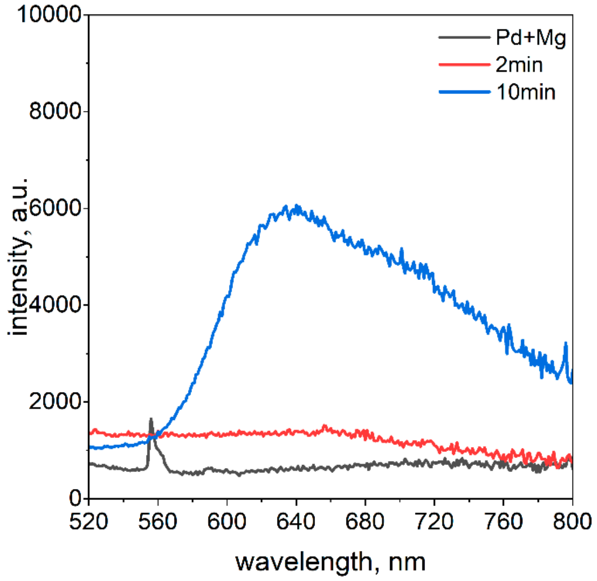
Pd+Mg
2min
10min
intensity, a.u.
0
2000
4000
6000
8000
10000
520
560
600
640
680
720
760
800
wavelength, nm
(h)

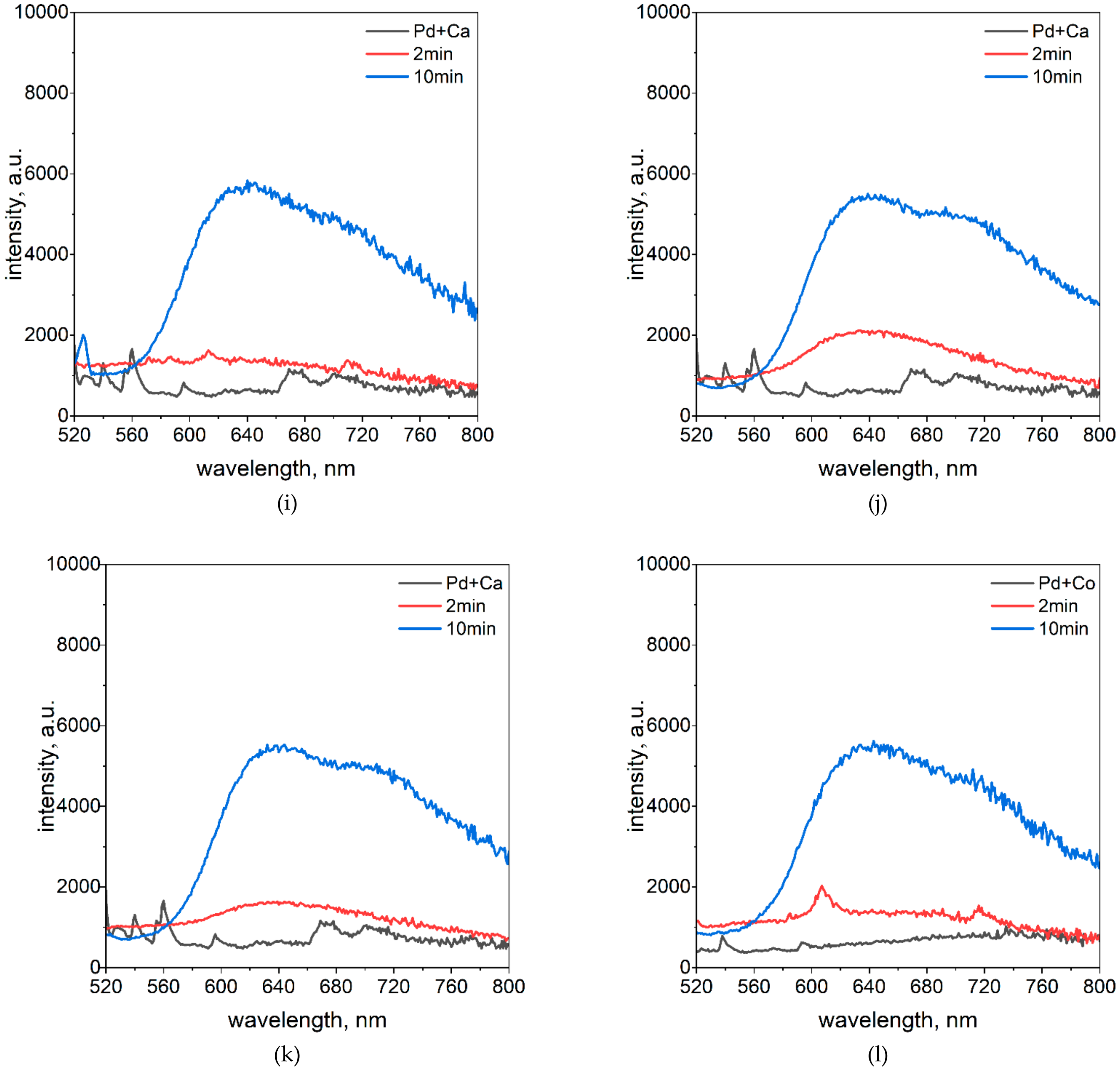

Figure S16. The evolution of fluorescence spectra of solutions containing Pd(II) with metal cations without TR OO (a), Pd(II), TR OO, and other metal cations—Li^+ (b), Na^+ (c), Na^+ (repetition 1) (d), Na^+ (repetition 2) (e), Al^{3+} (f), Ni^{2+} (g), Mg^{2+} (h), Ca^{2+} (i), Ca^{2+} (repetition 1) (j), Ca^{2+} (repetition 2) (k), and Co^{2+} (l)—at 2 min and 10 min after mixing in ethanol. Conditions: $C_{0,TR\ OO} = 1.25 \times 10^{-5}$ mol/dm^3, $C_{0,Pd(II)} = 3.75 \times 10^{-5}$ mol/dm^3, $C_{0,anions} = 0.00625$ mol/dm^3, volumetric ratio mixing of Pd(II) ions and TR OO = 3.0 mL: 1.0 mL, T = 50 °C.
